# SAR228810: an antibody for protofibrillar amyloid β peptide designed to reduce the risk of amyloid-related imaging abnormalities (ARIA)

**DOI:** 10.1186/s13195-018-0447-y

**Published:** 2018-11-28

**Authors:** Laurent Pradier, Véronique Blanchard-Brégeon, Andrees Bohme, Thomas Debeir, Jean Menager, Patrick Benoit, Pascal Barneoud, Véronique Taupin, Philippe Bertrand, Philippe Dugay, Béatrice Cameron, Yi Shi, Souad Naimi, Marc Duchesne, Marie Gagnaire, Tim Weeden, Tara Travaline, David Reczek, Leonard Khiroug, Mohamed Slaoui, Pascale Brunel, Hidehiro Fukuyama, Jeffrey Ravetch, Thierry Canton, Caroline Cohen

**Affiliations:** 1 Sanofi R&D Neuroscience Unit, Sanofi, 1Av P. Brossolette, 91385 Chilly-Mazarin, France; 2 Sanofi R&D Biotherapeutics Research, Vitry s/Seine, France; 3 Sanofi R&D Biotherapeutics Research, Framingham, USA; 4 Neurotar Ltd., Helsinki, Finland; 5 Sanofi R&D Preclinical Safety, Alfortville, France; 6 The Rockefeller University, New-York City, USA; 7 Present address: Dyne Therapeutics, Inc., 400 Technology Square, Cambridge, USA; 8 Present address: Laboratory for Lymphocyte Differentiation, RIKEN Center for Integrative Medical Sciences, Yokohama, Japan

**Keywords:** Alzheimer’s disease, Anti-Aβ immunotherapy, Protofibrillar Aβ peptide

## Abstract

**Background:**

Anti-amyloid β (Aβ) immunotherapy represents a major area of drug development for Alzheimer’s disease (AD). However, Aβ peptide adopts multiple conformations and the pathological forms to be specifically targeted have not been identified. Aβ immunotherapy-related vasogenic edema has also been severely dose limiting for antibodies with effector functions binding vascular amyloid such as bapineuzumab. These two factors might have contributed to the limited efficacy demonstrated so far in clinical studies.

**Methods:**

To address these limitations, we have engineered SAR228810, a humanized monoclonal antibody (mAb) with limited Fc effector functions that binds specifically to soluble protofibrillar and fibrillar forms of Aβ peptide and we tested it together with its murine precursor SAR255952 in vitro and in vivo.

**Results:**

Unlike gantenerumab and BAN2401, SAR228810 and SAR255952 do not bind to Aβ monomers, low molecular weight Aβ oligomers or, in human brain sections, to Aβ diffuse deposits which are not specific of AD pathology. Both antibodies prevent Aβ42 oligomer neurotoxicity in primary neuronal cultures. In vivo, SAR255952, a mouse aglycosylated IgG1, dose-dependently prevented brain amyloid plaque formation and plaque-related inflammation with a minimal active dose of 3 mg/kg/week by the intraperitoneal route. No increase in plasma Aβ levels was observed with SAR255952 treatment, in line with its lack of affinity for monomeric Aβ. The effects of SAR255952 translated into synaptic functional improvement in ex-vivo hippocampal slices. Brain penetration and decoration of cerebral amyloid plaques was documented in live animals and postmortem. SAR255952 (up to 50 mg/kg/week intravenously) did not increase brain microhemorrhages and/or microscopic changes in meningeal and cerebral arteries in old APPSL mice while 3D6, the murine version of bapineuzumab, did. In immunotolerized mice, the clinical candidate SAR228810 demonstrated the same level of efficacy as the murine SAR255952.

**Conclusion:**

Based on the improved efficacy/safety profile in non-clinical models of SAR228810, a first-in-man single and multiple dose administration clinical study has been initiated in AD patients.

**Electronic supplementary material:**

The online version of this article (10.1186/s13195-018-0447-y) contains supplementary material, which is available to authorized users.

## Background

Alzheimer’s disease (AD) is a progressive dementia, neuropathologically characterized by deposits of extracellular Aβ plaques and intraneuronal fibrillary tangles which contain hyperphosphorylated tau protein. Additional pathological changes in the brain include gliosis, inflammation, neuritic dystrophy, neuron loss, and changes in neurotransmitter levels [1].

Genetic evidence supports the key role of amyloid β (Aβ) peptide in the pathogenesis of AD: 1) all mutations causing familial AD increase the production of Aβ42 or the ratio of Aβ42 to the less aggregation-prone Aβ40 form [2]; and 2) genetic variants that reduce Aβ production lower the risk of developing AD [3]. However, anti-Aβ/amyloid strategies have not yet translated into an efficient and safe treatment for AD (reviewed in [4]). The recent interruption for futility of the phase III clinical study of BACE inhibitor verubescestat (Merck, MSD [5]) in prodromal AD subjects indicates that blocking the production of Aβ monomer is not efficacious at a disease stage when brain amyloid has been accumulating for at least a decade [6]. Quite differently, Aβ immunotherapy has the potential to neutralize or clear brain amyloid and therefore be of therapeutic value even later in the course of pathology progression than Aβ production inhibitors. However, after more than a decade of active development, encouraging clinical results have only recently been obtained. Immunotherapy failures thus far have been attributed in part to: 1) the dose severely limited by safety issues (vasogenic edema/microhemorrhage associated with amyloid-related imaging abnormalities (ARIA) reported in clinical settings, thus preventing efficacious brain levels being reached); 2) not targeting the appropriate toxic Aβ species; and 3) intervention late in the disease continuum. More promising results were recently generated with three antibodies binding to protofibrillar Aβ (aducanumab [7], gantenerumab [8, 9], and BAN2401 [10]) on biomarker endpoints that will need to be confirmed in larger ongoing clinical studies.

Aβ peptide can adopt multiple assembly forms and conformations which are operationally defined but with limited biophysical characterization, and include monomer, oligomer, soluble protofibrils of high molecular weight, and insoluble aggregated plaques (reviewed in detail in [11]). Which form of Aβ, if any single one, contributes to the disease remains controversial. From brain pathology studies, soluble oligomeric forms of Aβ (oAβ) have attracted attention since levels are correlated with AD dementia stage [12–14] and AD brain-derived soluble Aβ fractions lead to acute inhibition of synaptic plasticity and, over days, disruption of neurite integrity [15–18]. Synaptotoxicity is mediated by high molecular weight Aβ assemblies (oligomeric or protofibrillar forms) that continue to be further analyzed [19, 20]. Amyloid plaques and oligomers could possibly be intimately associated and in dynamic equilibrium, with evidence now suggesting that plaques could act as reservoir or as a nidus for Aβ oligomerization that could impair local synapses [21]. In this regard, inhibiting Aβ production (with a BACE1 inhibitor, for example) when amyloid plaques are already present would not prevent leakage of Aβ oligomers from plaques. The issue of which Aβ assembly to target is also quite important in view of the large differences in concentration of those species in the brain. Monomeric Aβ is abundant, with cerebrospinal fluid (CSF) concentrations of approximately 10 ng/ml (2.5 nM), and amyloid plaques can represent up to micromolar concentration of Aβ in brain tissue [22]. In comparison, soluble oligomeric and protofibrillar Aβ forms in CSF have been quantified in the low pM range with different detection techniques (approximately 3 pg/ml [23], 1 pg/ml [24], and below the limit of quantitation (LOQ) 6.25 pg/ml [14]; reviewed in [25]).

The different anti-Aβ monoclonal antibodies (mAbs) in clinical development have varying Aβ conformation binding profiles (reviewed in [4]). Solanezumab binds essentially to soluble monomeric Aβ; bapineuzumab and crenezumab bind to all Aβ conformation, while gantenerumab, BAN-2401, and the more recently described aducanumab bind to a large spectrum of oligomeric to fibrillar forms of Aβ, but with much lower affinity to monomeric Aβ. Precise differentiation among the latter three is quite limited due in part to the poor definition of the multiple Aβ oligomeric forms. In vivo, antibodies with limited differentiation among Aβ assemblies (bapineuzumab and crenezumab) would be preferentially engaged with the most abundant monomeric and insoluble Aβ fibrillar forms, leading to poor brain bioavailability to neutralize soluble oligomeric and protofibrillar forms. The monoclonal bioavailability issue in the brain is critical due to the detectable but very low brain penetration of therapeutic immunoglobulin Gs (IgGs) and is further exacerbated by the ARIA-related dose limitations.

In clinical trials, several anti-Aβ mAbs have elicited adverse events: vasogenic edema/microhemorrhage associated with amyloid-related imaging abnormalities (ARIA) in particular in ApoE4 carriers that are probably related to binding onto vascular amyloid, and local recruitment of an immune response via mAb Fc effector functions (reviewed in [26]). With regard to this point, crenezumab has been engineered on a human IgG4 Fc domain endowed with partially reduced effector functions leading to lower incidence of ARIA at higher doses than bapineuzumab [27], and exploratory post-hoc analysis suggested an effect on cognitive decline in an early AD subgroup. While deleterious regarding ARIA, the contribution of mAb effector functions to efficacy is quite controversial and it has been suggested that they were not necessary for in-vivo efficacy in animal models [28].

To address some of the aforementioned shortcomings of current Aβ mAbs, a humanized monoclonal antibody SAR228810 has been designed. Its key differentiating attributes are the specific pathology-associated antigen targeted soluble protofibrillar and fibrillar Aβ, and its drastically reduced effector functions (with two silencing mutations on a human IgG4 backbone) leading to improved benefit/risk balance. By targeting protofibrillar Aβ and not monomers, SAR228810 should be more effective in preventing plaque formation and oligomer-induced synaptic dysfunction and neurite loss than antibodies binding additionally to Aβ monomer. Here, we describe the in-vitro profile of SAR228810 (currently in phase 1 in AD patients) and its murine precursor SAR255952, a mouse monoclonal aglycosylated IgG1 antibody, comparing them with other protofibrillar Aβ antibodies gantenerumab and BAN2401 and their in-vivo activity compared with mouse bapineuzumab.

## Methods

### Antibody preparation

SAR255952 is an aglycosylated variant of Aβ protofibril-specific murine IgG1 antibody 13C3 [29] (patent application WO2009/065054) generated by mutation of the glycosylation site asparagine 297 to alanine in the Fc domain (N297A; position according to Kabat’s nomenclature). SAR228810 is a humanized version of SAR255952 with an engineered human IgG4 framework containing two amino-acid substitutions described to reduce half molecules (S241P) and effector functions (L248E [30]), as described in patent application WO2010/130946. An IgG1 mouse monoclonal antibody (Ctl-IgG1) against a nonmammalian protein was used as a negative control. The comparator is 3D6, the murine IgG2b version of bapineuzumab, directed against the N-term extremity of Aβ and insensitive to the assembly state of Aβ peptide [31].

#### Production and quality control

Antibodies were produced by transient transfection in human embryonic kidney cell line FreeStyle 293-F (Invitrogen) or from recombinant stable CHO cell lines. Briefly, cell culture supernatants were purified by a two-step protocol including affinity chromatography on protein A (MabSelect GE Healthcare) and CHT Ceramic Hydroxyapatite (Bio-Rad), and then formulated in phosphate-buffered saline (PBS; Dulbecco, reference 14,190–094) and sterile filtered (0.2 μm). Purity was estimated above 98% by SDS-PAGE under denaturing conditions and by size exclusion chromatography. The mass measured by LC/MS was in agreement with the amino acid sequence and the lack of N-glycans on the Fc domain for SAR255952 as expected. The apparent mass obtained by SDS-PAGE under nondenaturing conditions and by size exclusion chromatography was in agreement with the heterotetrameric structure of antibodies 150 kDa for SAR255952 and 160 kDa for the other glycosylated IgGs. For in-vitro studies, the following antibodies/batches were used: SAR228810 (C1024149), SAR255952 (batch 255,952-F04–015), BAN-2401 (batch VA214212), gantenerumab (batch VA214182), and their respective control isotypes: human IgG4 (hyb_CAA162_IgG4; batch VA112104), mouse Ctrl-IgG1 (hyb_CAA162_IgG1; batch VA213009), and human IgG1 (VA214053). For in-vivo studies the following antibodies and batches were used: SAR255952 (batch LP08190, control isotype Ctrl-IgG1 (hyb_CAA162_IgG1; batch LP09022), mouse 3D6-bapineuzumab (LP08030), and SAR228810 (LP09124). All batches were tested for sterility and lack of endotoxins.

### Synthetic Aβ preparations

Protofibrillar and low molecular weight (LMW) forms of Aβ1–42 were prepared as described previously [32]. Briefly, Aβ1–42 (Anaspec, reference 20,276) was dissolved in 10 mM NaOH at 100 μM and aliquots were stored at −80 °C. The sample was thawed on ice for 10 min and then diluted to 50 μM in NaCl/Pi buffer and incubated overnight at 37 °C. The sample was centrifuged at 17,900 g for 5 min at 16 °C and the supernatant was immediately collected. Protofibrils were separated from the LMW Aβ forms by size exclusion chromatography (SEC), and 500 μl of supernatant was loaded onto the Superdex 75 and fractions of 125 μl were collected. The concentration of protofibrillar (exclusion volume) and LMW fractions were determined by Bradford assay in reference to a standard curve established with peptide Aβ1–42.

### Preparation of fibrils from Aβ, IAPP, or α-synuclein

For Aβ1–42 fibrils, Aβ1–42 (1 mg) was dissolved in 200 μl 10 mM NaOH (5 mg/ml). Aliquots (100 μl) were diluted in 400 μl PBS 1.25× (final concentration 1 mg/ml) and were incubated at 37 °C for 72 h.

For islet amyloid polypeptide (IAPP) fibrils, 1 mg IAPP (Anaspec, reference 60,804) was dissolved in 200 μl dimethylsulfoxide (DMSO) 50% (5 mg/ml). Aliquots of 100 μl were diluted in 400 μl PBS 1.25×. Samples were incubated at 37 °C for 72 h.

For α-synuclein fibrils, α-synuclein (Millipore, reference AG938) was provided lyophilized in Tris-HCl buffer, pH 7.4, and 500 μg α-synuclein were dissolved in 200 μl H_2_O MilliQ + 50 μl 500 mM NaCl solution. Samples were incubated by shaking at 37 °C for 7 days (750 rpm).

After the incubation, samples were centrifuged at 17,000 g for 20 min at 4 °C. The supernatants were discarded. Pellets of synuclein fibrils were washed twice with 200 μl PBS and then resuspended in 100 μl PBS, and the fibril sample concentration was determined using the Bradford test.

### Binding assay by ELISA

Wells of enzyme-linked immunosorbent assay (ELISA) plates were coated with 50 μl protofibrils, LMW Aβ, or fibrils at 1 μg/ml in PBS. Plates were incubated overnight at 4 °C. Antigen excess was eliminated, and wells were blocked with 200 μl/well PBS-T (PBS + Tween 0.02%)/milk 5% for 2 h at room temperature. Wells were washed four times with 300 μl PBS-T, and 50 μl test antibody was added to wells after serial threefold dilution in PBS-T starting from a concentration of 25 μg/ml. Plates were incubated for 1 h at room temperature and then washed four times with 300 μl/well PBS-T. Secondary antibody (rabbit anti-mouse or goat anti-human) conjugated with horseradish peroxidase (HRP) diluted to 1/10000 in PBS-T was added and incubated for 1 h at room temperature. Wells were then washed four times with 300 μl PBS-T, and 100 μl/well of 3,3′,5,5′-tetramethylbenzidine (TMB) was added and incubated for 10 min. The reaction was stopped by the addition of 100 μl stop solution. Plates were read to measure the optical density (OD) at 450 nm. Dose-response curves with 12 concentrations in duplicate were performed to derive the EC50s.

### Surface plasma resonance (SPR) affinity determination

Analysis was performed on a Biacore T100 with HBS-EP+ (10 mM HEPES, 150 mM sodium chloride, 3 mM EDTA, and 0.005% P20) as the running buffer. A Series S Protein A sensor chip was used for the analysis. Antibodies were diluted to 5 μg/ml in HBS-EP+. Monomeric Aβ1–42 peptide (SEC-purified) was diluted to 1000 nM (4.5 μg/ml) in HBS-EP+ and then serially diluted twofold for a total of six concentrations. Aβ1–42 protofibrils were diluted to 100 nM (66 μg/ml) in HBS-EP+ and then serially diluted twofold for a total of six concentrations. Antibodies were captured via Fc domain on the Protein A surface for 60 s at 10 μl/min and then the Aβ peptide solution (monomeric or protofibrils) was injected over the surface for 180 s at 30 μl/min followed by a 360-s dissociation. The Protein A surface was regenerated with a 60-s injection of 10 mM glycine-HCl, pH 1.7. The resulting sensorgrams were double-referenced and fitted to a 1:1 binding model to determine k_a_, k_d_, and K_D_.

### Characterization of binding to human AD brain sections

#### Digoxigenin conjugation of primary human mAbs

To characterize binding patterns of human mAb SAR228810 in human tissues, the primary antibodies (SAR228810, isotype controls, and 3D6 mAbs) were conjugated with digoxigenin (dig). Briefly, DIG-NHS (11,333,054,001, Roche) at 2 mg/ml was diluted in DMSO. Then, 23.5 μl (70 nmol) of this solution was added to 1 mg antibodies in 1 ml PBS (pH 8.5). This mixed solution was incubated for 2 h under gently stirring at room temperature. The labeled protein was eluted in PBS through Sephadex G-25 columns (11,418,165,001 Roche). The initial titration of labeled protein was determined with Bradford reagent and measured by FloStar (Optimal). Samples with a concentration of labeled protein over 100 μg/ml were pooled and concentrated on an Amicon ultra-15 centrifugal filter system (UFC910008, Millipore). The SAR228810-dig concentration was 1.31 mg/ml, huIgG4-dig isotype 0.73 mg/ml, and 3D6-dig 0.71 mg/ml.

#### Human brain tissue sections

Formalin-fixed and paraffin-embedded (FFPE) human AD and controls cortices sections were from internal collection. The diagnosis of the cases was given based on clinical and hospital neuropathologist’s findings, and by internal quality control of senile plaques and neurofibrillary tangles before usage. Two AD and two control donors were evaluated in this study.

#### Immunohistochemistry (IHC) procedure

FFPE sections were dewaxed by xylene and dehydrated with progressive ethanol (70%, 95%, and 100%) until distilled water. A pretreatment was performed with 70% formic acid for 10 min and rinsed under running water for 5 min before the IHC procedure. IHC was performed using the Ventana Discovery XT automated System (Ventana Medical System, Inc.) with a standard protocol. All detection systems were manufactured by Ventana Medical System Inc. IHC optimal concentrations for each antibody were determined by individual pilot studies under the standard condition. The primary antibody (10 μg/ml SAR228810-dig or IgG4 isotype control-dig, 5 μg/ml 3D6-dig) was incubated on tissue sections for 4 h, and then 2 mg/ml secondary mAb (IgG1 murine anti-dig-biotin, B7405; Sigma) for 40 min. The tertiary mAb used UltraMap™ anti-mouse conjugated with either alkaline phosphatase or horseradish peroxidase incubated for 16 min. Sections were revealed either by DAB (bright-field light microscope) or Fast red (fluorescence light microscope detection systems).

#### Congo red dye (fibrillary plaque marker) protocol

Dewaxed sections were stained with Congo red solution (Microscopy Congo red staining kit, 101,641; Merck) for 10 min, rinsed in distilled water, and differentiated in KOH for 30 s. After dehydration in 95% and 100% ethanol, the sections were cover-slipped using mounting media (Cytoseal 60, 8310–4; Richard-Allan Scientific Inc.).

#### Analysis method

Fast red and Congo red dye sections were analyzed by a fluorescence microscope (BX61; Olympus) and photographed by the cell F software system (Olympus). DAB-immunolabeled sections were examined by a bright-light microscope (Eclipse E400 4; Nikon) and imaged by a digital image acquisition system (NIS-Elements F 3.0; Nikon).

### Primary neuronal culture oAβ42 toxicity assays

Primary neuronal cultures were prepared from the brains of 16-day-old mouse (OF1, Charles River Laboratories, France) embryos by dissecting and then dissociating cerebral cortices. Cells were plated in Dulbecco’s modified Eagle’s medium (DMEM) supplemented with N2 and B27 at a cell density of 4 × 10^5^ cells/ml in poly-d-lysine-coated 96-well culture microplates.

On the day of experiment, the concentrated working solution of all antibodies and control isotypes was prepared as a 10× solution by diluting each stock solution to 1.5 mg/ml in DMEM. Lower tested concentrations were prepared by diluting the concentrated working solution in DMEM. Final concentrations of tested antibody within the culture wells were 150, 50, or 15 μg/ml, corresponding to 1, 0.33, or 0.1 μM, respectively.

Aβ42 oligomers (oAβ42) were prepared as previously reported [33]. Briefly, human Aβ42 peptide (reference 641–15; California Peptide Research Inc., USA) was first solubilized at 1 mM in hexafluoroisopropanol (HFIP; Sigma H8508). The resulting solution was aliquoted in microcentrifuge tubes (VWR 20170–293), the HFIP was allowed to evaporate in a ventilated hood, and the resulting clear peptide film was dried under vacuum in a SpeedVac and stored desiccated at −20 °C under vacuum. On the day of experiment, the frozen Aβ42 sample was resuspended at 5 mM in DMSO (Sigma D2650). Aβ42 oligomers were obtained by diluting the 5 mM Aβ42 working solution to 100 μM in ice-cold cell culture medium and incubating for 1 h at room temperature. This oAβ42 preparation was added to the culture medium at a final concentration of 5 μM. After 6 days in vitro, primary neuronal cultures were pretreated with anti-Aβ antibodies for 1 h in culture medium without B27. Then the neurons were incubated with oAβ42 (5 μM) for 48 h. Control application of drug-free medium but supplemented with the corresponding DMSO concentration (i.e., 0.1%, the same as for oAβ42 treatment) was run in parallel to the application of the test substance.

#### Quantification of caspase-3/7 enzymatic activity

Following experimental treatment, caspase-Glo 3/7 Assay kit solution (Promega Corporation USA, G7790) was mixed to each well and incubated for 4 h at room temperature. After incubation, fluorescence in samples was quantified using a Spectramax Gemini plate reader. Fluorescence intensity is proportional to caspase-3/7 enzymatic activity.

#### Quantification of neurite network

Following experimental treatment, cell cultures were fixed with 4% formaldehyde solution (Merck, 100,496) and then permeabilized in PBS/0.2% Triton X-100 for immunostaining. Permeabilized cells were incubated overnight at 4 °C with a primary antibody recognizing the neuronal protein marker microtubule-associated protein 2 (MAP2; Millipore AB5622) followed by an incubation with a secondary antibody corresponding to Alexa Fluor 594 coupled-anti-rabbit antibody (Molecular probes, A11012) for 1 h at room temperature. After an additional washing step, fixed cells were then incubated for 15 min with Hoechst 33342 (Molecular probes H3570) to stain for nuclei (0.2 μg/ml). Analysis of the neurite network was performed on MAP-2 immunostained and Hoechst-stained cells using automated high content image analysis (In Cell Analyzer 2200, GE Healthcare, UK). The measurement of the neurite area (expressed as μm^2^) was used as the neurite network index.

### In-vivo pharmacological studies with murine SAR255952 mAb

#### Animals

Animal used were males from the APPSL transgenic line (expressing the 751 amino-acid variant of APP harboring the so-called “Swedish” (K670 N/M671 L) and “London” (V717F) mutations under a Thy1.2 promoter) as described in reference [34] and backcrossed to C57Bl/6 mice, and age- and sex-matched littermate wild-type mice. Animals were bred and maintained in Charles River France facilities and supplied at the age of 2 months (weight ranging from 21 g to 26 g) for pharmacological studies. Experiments were performed at Sanofi in an AAALAC-accredited facility in full compliance with the standards for the care and use of laboratory animals, according to French and European Community (Directive 2010/63/EU) legislation. All procedures were approved by the local Animal Ethics Committee (CEEA #24) and the French Ministry for Research. Mice were housed individually in an enriched environment in a pathogen-free facility at a constant temperature of 22 ± 2 °C and humidity (50 ± 10%) on a 12-h light/dark cycle with ad libitum access to food and water. Animals were randomized to the different groups. All analyses were performed with operators blinded to the treatment groups.

#### Treatments

Male APPSL mice (*n* = 15 per group) were treated once a week by intraperitoneal (i.p.) administration of antibodies at the following indicated doses dissolved in PBS under a volume of 10 ml/kg: 10 mg/kg (Ctl-IgG1); 1, 3, and 10 mg/kg (SAR255952); and 3 and 10 mg/kg (3D6). All treatments started at the age of 2 months. Treatment duration was 20 weeks for the biochemical and histological readouts. For the electrophysiological studies, four groups of mice treated with Ctl-IgG1 (10 mg/kg), SAR255952 (3 or 10 mg/kg), or 3D6 (10 mg/kg) and a group of wild-type controls were maintained on extended treatment up to the age of 9 to 10 months when they were individually processed for brain slice preparation and electrophysiological recordings. Mice were anesthetized by intravenous (i.v.) coadministration of xylazine (2.7 mg/kg) and ketamine (87 mg/kg) under a volume of 10 ml/kg and were decapitated. Brains were removed, and one hemi-brain was prepared for biochemical quantitative measurements and the other was used for immunohistochemistry. Due to spontaneous mortality in this transgenic strain, 9–12 mice survived until the end of the treatment period with no group bias. All mice were used for biochemical analysis and are reported as individual values, while immunohistochemistry analysis was performed on nine mice per treatment group.

#### Biochemical analysis

For biochemical quantitative measurements, the hemi-cortex was prepared for quantitative measurement of total Aβ accumulation and the hemi hippocampus for mRNA preparation. For biochemical analysis of total Aβ levels, the hemi-cortex was weighed and homogenized in 10 volumes (w/v) in ice-cold buffer solution (composed of 0.32 M sucrose, Tris-HCl 4 mM, pH 7.4, and containing a cocktail of protease inhibitors (Complete™, Roche Diagnostics GmbH, Manheim-Germany)). One milliliter of homogenate was then mixed with 2 ml of a 9 M guanidine hydrochloride (GH) solution in 50 mM Tris-HCl, pH 7.4, and mixed by three 15-min bath sonications (total duration of the cycle 1 h), followed by centrifugation at 50,000 g at 4 °C for 120 min. The supernatant was retrieved and called as guanidine extracts (“total Aβ”). Samples were further diluted 1:1000 with ice-cold saline buffer containing 150 mM NaCl, 0.5% bovine serum albumin (BSA; w/v), 0.05% Tween 20 (w/v), and 20 mM Tris-HCl, pH 7.6. Aβ standards (Bachem) were prepared with the same final GH concentration. For one experiment, Tris-soluble, membrane, and insoluble fractions were prepared by differential extraction following reported procedures [35]. The final pellet was solubilized in 50 mM Tris-HCl, pH 7.4, 6 M GH.

Immunoblot analysis using the APP antibody 22C11 indicated that SAR255952 did not affect brain APP levels (data not shown).

##### Aβ quantification

Aβ peptides were detected by electrochemiluminescence assays using anti-Aβ antibodies and a Sector Imager 2400 Analyzer (Meso Scale Discovery, MSD, Gaithersburg, MD, USA) as previously described [34] with minor modifications. The 4G8 murine monoclonal antibody (Senetek PLC), which specifically recognizes an epitope within the 18–24 amino-acid region of Aβ, was ruthenylated with MSD Sulfo-TAG NHS ester, according to the supplier’s instructions (MSD). It was thereafter used in conjunction with a 6E10-biotinylated murine monoclonal antibody (Senetek PLC), which binds to an epitope within the 2–11 amino-acid region of Aβ. This assay can detect both Aβ40 and Aβ42 peptides and all shorter forms with a core region comprised between position 2 and 24 of Aβ, thereafter termed pan-Aβ. To specifically measure Aβ42 levels, 6E10 was replaced by a murine monoclonal antibody, 22F9, which binds to the C-terminus of Aβ42 (with no cross-reaction with Aβ40). The levels of Aβ peptide were expressed as μg/g of wet tissue weight. For quantification of the soluble and insoluble fraction, the Aβ panel kit (MSD; reference K15200G) was used to quantify Aβ38, Aβ40, and Aβ42 subspecies and values are reported normalized to the protein content of the initial homogenate.

##### Antibody quantification in body fluids

ELISA assays with coated fibrillary Aβ were used to dose serum and CSF levels of antibodies as described in the section above, using a calibration curve in parallel with recombinant SAR255952 or 3D6 antibodies.

##### RNA analysis

Hemi-hippocampus from each mouse was placed in a round-bottomed 2-ml tube containing a stainless magnetic bead and 0.5 ml of Applied Biosystems nucleic acid purification lysis solution (1×). The tissue was homogenized using a Mixer Mill MM 300 (Retsch) during 2 × 1.30 min at 20 Hz. Total RNA was isolated using the 6100 PrepStation (Applied Biosystems), according to the manufacturer’s instructions, including a DNase treatment (protocol Isolation of Total RNA from Plant and Animal Tissue). To assess the quality and concentration of the total RNA, 1 μl was analyzed on an RNA LabChip (Agilent) using a 2100 Bioanalyser (Agilent Technologies).

For real-time polymerase chain reaction (PCR), 2 μg total RNA from each mouse was reverse transcribed with oligo (dT)16 and random primers using a High-Capacity cDNA Archive Kit (Applied Biosystems), following the manufacturer recommendations. The final reverse-transcription reaction-included template was 100 μl. Samples were then incubated for 10 min at 25 °C, followed by 120 min at 37 °C, and then heated at 95 °C to denature the enzymes and stop the reaction. For real-time PCR, cystatin F was amplified using commercial Taqman probes (Mm00438349_m1) and the housekeeping gene R.L37A was amplified using a Quantitech primer assay from Qiagen (QT00252266). PCR amplification was performed according to the manufacturer’s instructions (Applied Biosystems) using an ABI Prism 7900 sequence detector.

#### Histology

Subsequent to immersion fixation in 4% formaldehyde, frozen hemi-brains were cut serially along the entire latero-medial axis into sagittal sections (Cryostat HM560, Microm). Randomly selected series of 30 μm-thick free-floating sections were used to perform Aβ peptide and IgG1 immunohistochemistry, and respectively used to quantify cerebral Aβ peptide deposition and to detect therapeutic antibody that enters cerebral tissue. After pretreatment with 80% formic acid for 3 min for Aβ immunostaining only, sections were incubated at room temperature for 30 min in 0.3% hydrogen peroxide and finally in blocking buffer (TBS with 0.2% BSA, Sigma). They were incubated overnight at 4 °C with primary antibodies: biotinylated mouse monoclonal anti-Aβ peptide antibody (human Aβ17–24 (4G8, reference 9240–10, Signet, dilution 1/200) or biotinylated goat anti-mouse IgG1 antibody (reference 1070–08, SouthernBiotech, dilution 1/200). Sections were further incubated with peroxidase-coupled avidin complex (Vectastain ABC kit, Vector Laboratories, dilution 1/400) for 30 min. Diaminobenzidine substrate was used for color development. Sections immunostained with IgG1 were counterstained using 0.2% Congo red solution in NaCl-saturated 80% ethanol (Accustain amyloid stain Congo Red Kit, HT60, Sigma).

##### Image analysis

For Aβ immunostaining, images of the entire sections were acquired on an Olympus dotslide BX scanner and quantitatively analyzed on a computer-based workstation (Mercator system/Explora Nova using Dotslide software). Based on a thresholding procedure allowing for automatic detection of individually stained deposits within both hippocampal and cortical areas, the total detectable surface area (μm^2^) over eight anatomical levels representative of the entire latero-medial axis of the hemi-brain was measured. For IgG1/Congo double-staining, qualitative image analysis was performed on an Olympus scanner using bright-field microscopy.

### Electrophysiology

On each day of experimentation, a mouse from one of the five extended treatment groups in interleaved order was processed for brain slice preparation and ex-vivo recording of glutamatergic field potentials in the hippocampus following classical methods. Briefly, the mice were culled by cervical dislocation and the brain was quickly dissected out and cooled in artificial cerebrospinal fluid (ACSF: 124 mmol/l NaCl, 3 mmol/l KCl, 1.3 mmol/l MgSO_4_, 2 mmol/l CaCl_2_, 1.3 mmol/l NaH_2_PO_4_, 26 mmol/l NaHCO_3_ and 10 mmol/l glucose). Coronal slices (350 μm thick) were cut with a vibratome (Integraslice 7550 PSDS, Campden Instruments) and placed in a submersion-type recording chamber (Warner RC-26G; volume 200 μl) through which warmed (28–30 °C) and gassed (95% O_2_/5% CO_2_) ACSF was continuously superfused at 3 ml/min. The healthiness of hippocampal synaptic function was assessed by analyzing the excitatory pathway projecting from the CA3 to the CA1 area. Electrical stimulations (0.1 ms in duration) were delivered through bipolar tungsten electrodes placed on the trajectory of the Schaeffer collaterals. Extracellular field excitatory postsynaptic potentials (EPSPs) were recorded in the CA1 stratum radiatum through low impedance glass micropipettes filled with ACSF. Recordings started with a baseline stabilization period for at least 1 h during which the size of the EPSPs elicited by supraliminal stimulations delivered every 30 s was monitored. Once stabilized, a sequence of 16 stimuli of graded intensity from 25 μA to 400 μA with a step of 25 μA was delivered to each preparation. Stimulation/response curves were constructed by plotting the mean (± standard error of the mean (SEM)) EPSP amplitudes against the stimulation intensities applied to the slice at each of the 16 predefined intensity levels.

#### Two-photon in-vivo imaging

One 8-month-old APPSL female mouse was anesthetized with an i.p. injection of ketamine (100 mg/kg) + xylazine (10.0 mg/kg). A cranial window over the right cortical hemisphere was surgically implanted as described previously [36]. Methoxy-X04, a widely used tracer of amyloid deposits [37], was injected i.p. 24 h before the first imaging session (10 mg/kg, 5 mg/ml solution). Stacks of images were collected with the vertical step of 2 μm. The SAR255952 antibody labeled with a fluorescent dye (DyeLight-649, Cambridge UK) was injected i.v. (20 mg/kg, 4.7 mg/ml) during the first imaging session. Four additional imaging sessions were performed at 30 min, 24 h, 72 h, and 144 h after the i.v. administration of the fluorescently labeled SAR255952.

In-vivo two-photon imaging was performed using the FluoView1000 MP system (Olympus Europa, Hamburg, Germany) equipped with a mode-locked Ti:Sapphire Mai-Tai DeepSee femtosecond laser (Spectra-Physics, Santa Clara, CA, USA) and a water immersion 25× objective XLPlan N with high numerical aperture (NA = 1.05) specially designed for in-vivo imaging (Olympus Europa, Hamburg, Germany). The laser wavelength of 800 nm was used for excitation of both Methoxy-X04 and DyeLight-649. Fluorescence was collected using a beam-splitter and two band-pass filters: 420–500 nm for Methoxy-X04 and 660–740 nm for DyeLight-649.

Vertical stacks of square images (500 × 500 μm^2^) were collected with the vertical step of 2 μm (total imaged depth of stacks: 350–400 μm) at the temporal interval of 10–30 min. Each imaging field was imaged repeatedly in every imaging session. Images were preprocessed for noise reduction using ImageJ/Fuji plugins (Median 3D and Gaussian Blur 3D). All detected plaques were visually verified at each time point, and only the plaques > 20 μm in diameter were analyzed. A custom-made MATLAB (Mathworks, Natick, MA, USA) routine was used to align the coordinates of the plaques imaged at the different time points. Regions of interest (ROI) were carefully drawn to include five parenchymal plaques, five perivascular amyloid deposits, five intravascular regions, and five parenchymal regions. ROIs included the parenchymal plaques located 30 to 90 μm below the cortical surface. The ROI coordinates were stored, and measurement of fluorescence intensity in the antibody channel was performed for all ROIs in all subsequent image stacks.

### Induction of immunotolerance for testing of human antibodies in mice

The anti-CD4 monoclonal antibody GK1.5 was produced from a hybridoma acquired from American Type Culture Collection, ATCC® TIB-207 [38, 39]. For immunodepletion induction, 11- to 12-week-old APP751SL mice were treated at day 0 and day 2 by intraperitoneal injection of 150 μg GK1.5 while a control group (i.e., non-immunotolerized mice) was administered with PBS. On day 4, blood was taken by retro orbital sampling on a heparin-lithium coated tube (microvette®, Sarstedt AG, Germany). CD4+ T lymphocytes were quantified using FACS analysis on a FACSCantoII flow cytometer using standard protocols with CD45-FITC (clone 104 BD Pharmigen), CD3e-alexa Fluor 647 (clone 17A2, eBioscience), and CD4-PE (RM4–4 clone, BioLegend) antibodies. All GK1.5-treated animals displayed reduced CD4 as evidenced by a ratio of CD4+ lymphocytes/total CD3+ lymphocytes of 0.01 ± 0.01 in the treated group compared with 0.48 ± 0.20 in the untreated mice.

#### Chronic treatments with human therapeutic antibodies

SAR228810, SAR255952, and control Ctl-IgG1 antibodies were administered at 10 mg/kg (weekly i.p. for 19 weeks, initiated 2 days after the second GK1.5 administration) in immunotolerized male APPSL mice starting at 3 months of age (*n* = 13–14 per group). A control group of non-immunotolerized APPSL mice (i.e., not treated with GK1.5 antibody) was treated with Ctl-IgG1 and used as a control for the immunodepletion induction. Mice were culled at 7 months of age. Quantitative assessment of preventive activity against cerebral Aβ peptide deposition was performed using histology and RNA analysis as described above (for each group, animal numbers are indicated in Table 4).

### In-vivo safety study

To determine the potential toxicity of SAR255952, experiments were carried out in older transgenic APPSL female mice (12 to 13 months of age at initiation of dosing, *n* = 13 to 16 per group at the start) which develop higher levels of amyloid plaques and congophilic angiopathy than males. Mice received PBS vehicle for the control group or SAR255952 at 10, 20, or 50 mg/kg i.v. administration by bolus injection (tail vein) once weekly for 9 weeks. A separate group of female mice (*n* = 13 at initiation of dosing) received 3D6 (the positive control group) at 40 mg/kg i.v. administration by bolus injection (tail vein) once weekly for 9 weeks. Gender and age were selected to insure abundant parenchymal and vascular amyloid load and a high antibody dose was used to maximize potential for brain microhemorrhages as previously reported [40] and from an initial in-house study.

Various parameters were evaluated, including mortality, clinical signs, body and organ weights, food consumption, hematology, and clinical chemistry.

#### Anatomic pathology

Mice were anesthetized with isoflurane for subclavicular clinical pathology blood sampling and then euthanized by exsanguination from the abdominal aorta. Terminal body weight was obtained on the day of necropsy and the absolute and relative (organ to body) weights were determined. A thorough necropsy (including abdominal and thoracic cavities) was performed and macroscopic observations were recorded. A comprehensive list of tissues/organs were collected, preserved in 10% neutral-buffered formalin, and then processed to paraffin blocks. Tissue blocks (except the brain, see below) from control and SAR255952 high-dose groups were sectioned, stained with hemalum, eosin and saffron (HES) according to standard protocols, and evaluated by light microscopy. For the brain, five equidistant samples through the entire organ were made to obtain a total of six coronal brain sections that were embedded anterior side down. For each brain, two coronal brain sections were embedded per block resulting in the preparation of three paraffin blocks per brain. All brain blocks from all groups were sectioned. The three brain blocks per animal were stained with HES and with Perl’s, resulting in six HES-stained and six Perl’s-stained coronal brain sections per mouse. In the control, SAR255952 high-dose, and 3D6 groups, six additional sections at 50-μm intervals per block were performed and stained with Perl’s, resulting in 36 additional coronal brain Perl’s-stained sections per mouse. All stained brain sections were examined by light microscopy.

### Expression of results and statistical analysis

Mean ± SEM in-vitro EC50 values for antibody binding affinities were calculated by nonlinear regression according to a classical four-parameter logistic model (GraphPad Prism 7.02).

For other biochemical data, results are expressed as mean ± SEM (Tables 1–3). For quantitative PCR (qPCR), cycle threshold (Ct) values were converted in relative quantity (RQ) using the equation: RQ equal to 2(Ct(L37a) – Ct(CystatinF)) for each hippocampus sample divided by 2(Ct (L37a) – Ct(CystatinF)) for a referent total brain sample.

**Table 1:** In-vitro binding characteristics of SAR2559952, humanized SAR228810, and comparators

Mouse IgG	ELISA Aβ		ELISA aggregated proteins				
	EC50 ng/ml		EC50 ng/ml				
Mean EC50	Protofibril (soluble)	LMW/monomer	Fibril/aggregates			IAPP fibrils	Synuclein fibrils
SAR255952	36 ± 5.5	3751 ± 130	68 ± 15			NB up to 100 μg/ml	NB up to 100 μg/ml
3D6	61 ± 4.4	86 ± 6	45 ± 17			NB up to 100 μg/ml	NB up to 100 μg/ml
Human IgG	ELISA Aβ		ELISA aggregated proteins			SPR	
	EC50 ng/ml		EC50 ng/ml			K_d_ (M)	
	Protofibrils (soluble)	LMW/monomer	Aβ fibrils	IAPP fibrils	Synuclein fibrils	Aβ monomer	Aβ protofibrils (soluble)
SAR228810	37 ± 2	14,403 ± 3403	16.7 ± 3.4	NB	NB	NB up to 1000 nM	4.3 ± 5 × 10^–10^
3D6 human IgG	32 ± 2	NA	7.7 ± 2.2	NA	NA	1.7 ± 1 × 10^–8^	< 10^–10^
Gantenerumab	26 ± 8	NA	19.9 ± 4.7	NA	NA	3.4 ± 3 × 10^–5^	8 ± 3 × 10^–10^
BAN-2401	21.3 ± 9	NA	15.5 ± 5.1	NA	NA	8.7 ± 4 × 10^–6^	< 10^–10^

Binding experiments performed three to five times, mean ± SEM EC50 values are presented

*Aβ* amyloid β, *ELISA* enzyme-linked immunosorbent assay, *IAPP* islet amyloid polypeptide, *LMW* low molecular weight, *NA* not available, *NB* no binding,

For immunohistochemistry, the average of individual measurements of total surface occupied by Aβ immunostaining over eight sections was used to calculate group means. Normality and homogeneity of variances hypotheses were tested with Shapiro-Wilks and Levene tests, respectively. Although the Aβ42 was not normally distributed in the 3D6 (10 mg/kg) group due to an extreme value, parametric analyses were used. Data were assessed with one-way analysis of variance (ANOVA), followed by post-hoc two-sided Dunnett’s test versus control. The Ctl-IgG1 group was first compared with the SAR255952-treated groups and then to the 3D6-treated groups. For cystatin F mRNA expression, a nonparametric analysis was used since, in most of the cases, the hypotheses were not fulfilled. mRNA data were assessed with a nonparametric Kruskal-Wallis test, followed by a post-hoc two-sided Kruskal-Wallis multiple comparisons test versus control. The statistical analyses were performed using the SAS system release 8.2 for SUN4 via Everstat 5.0 software. A probability value of *p* < 0.05 was considered significant.

The amplitudes of the EPSPs were subjected to two-way ANOVA with stimulation intensity as the repeated factor. Post-hoc analysis of effects at each level of stimulation intensity was performed by Dunnett’s test comparing the Ctl-IgG1 group with each dose of SAR255952. Results are expressed as mean values ± SEM in the figures.

## Results

**Fig. 1:**
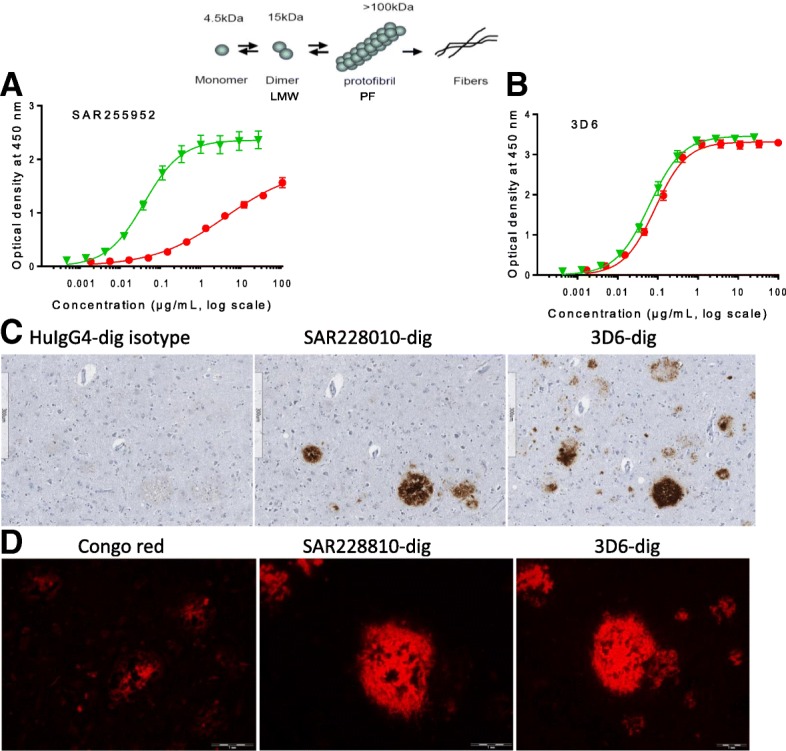
Specificity of SAR255952 and humanized SAR228810 for soluble Aβ protofibrils and fibrillar forms but not low molecular weight Aβ or monomers. Synthetic Aβ_42_ was allowed to aggregate in aqueous saline buffer. Soluble, low molecular weight (LMW) Aβ complexes and Aβ monomers (red circles) were separated from higher order fibrillar aggregates (green triangles) by size exclusion chromatography. In ELISA apparent affinity determinations, SAR255952 demonstrates high affinity and strong preference for protofibrillar Aβ versus monomeric Aβ (**a**), unlike 3D6 (bapineuzumab) that equally binds to both Aβ conformations (**b**). Similar data were obtained with the humanized version SAR228810. In human AD cortical sections, SAR228810 specifically binds to compact amyloid deposits while 3D6, in addition, detects a large number of diffuse deposits (chromogenic stain panel **c**). This was confirmed with fluorescence labeling with SAR228810 binding to a large area around Congo red-positive core plaques, while 3D6 labeled in addition non-Congo red-positive deposits (diffuse deposits). Symbols and error bars indicate mean ± SEM from ELISA readings. Scale bars represent 300 μm in panel **c** and 1 μm in panel **d**

SAR255952 is a mutant aglycosylated version of Aβ protofibril-specific murine antibody 13C3 ([29]; WO2009/065054) obtained by mutation of the glycosylation site in the Fc domain (N297A). SAR228810 is the corresponding humanized version designed with a human mutated IgG4Fc domain. As designed, both antibodies display reduced binding to FcγR and complement C1q (Additional file 1: Table S1), indicating drastically reduced effector functions. In the present study, we first confirmed by ELISA that SAR255952 maintained a higher affinity for soluble HMW Aβ protofibrils (size exclusion chromatography purified) compared with LMW Aβ complexes (including monomers) with EC50 values of 36 ± 5.5 ng/ml (*n* = 6) and 3751 ± 130 ng/ml (n = 6), respectively, while the pan Aβ-conformer antibody 3D6 (murine bapineuzumab) displayed similar affinities for both Aβ conformations (Fig. 1 and Table 1). We further demonstrated that SAR255952 was specific for Aβ fibrils (EC50 value of 68 ± 15 ng/ml) with no binding to IAPP or α-synuclein fibrils (Table 1). The Aβ peptide conformation-restricted binding was further confirmed by Aβ peptide fragment scan analysis that failed to detect high affinity binding of SAR255952 to any linear 12 amino-acid peptide from the Aβ sequence (data not shown).

The humanized version SAR228810 displayed a similar specificity for Aβ protofibrils versus monomeric Aβ as confirmed by ELISA, with EC50 values of 37 ± 2 ng/ml (*n* = 4 separate experiments, in quadruplicate each) versus 14,403 ± 3403 ng/ml (n = 6), respectively (Table 1). Using surface plasma resonance (SPR), SAR228810 displayed high affinity for Aβ protofibrils (K_D_ = 4.3 ± 5 × 10^−10^ M) and no binding for monomeric Aβ up to 1000 nM, while 3D6-human chimera had high binding affinity for both Aβ conformers (K_D_ < 10^−10^ M (resolution limit of this technique) for Aβ protofibrils and K_D_ = 1.7 ± 1 × 10^−8^ M for monomeric Aβ (Table 1)). Again, SAR228810 was specific for Aβ aggregated forms versus IAPP or synuclein aggregates. Two additional clinical anti-protofibrillar Aβ antibodies, gantenerumab and BAN2401, also displayed high affinity for fibrillar and protofibrillar Aβ but had residual binding to monomeric Aβ (K_D_ = 3.4 × 10^−5^ M and 8.7 × 10^−6^ M, respectively).

### Characterization of SAR228810 binding in human brain sections

The validation of SAR228810-dig mAb was carried out in human AD and control cortices. Using a chromogenic method, SAR228010-dig was shown to specifically bind Aβ deposits in the human AD cortex (Fig. 1c), without any binding in the normal brain (not shown). In adjacent AD brain sections, 3D6-dig labeled a higher number of Aβ deposits with various morphological patterns in comparison with SAR228010-dig. As a control, the huIgG4-dig isotype did not show any labeling (Fig. 1c).

To characterize the binding difference between the two antibodies, we compared the SAR228010-dig and 3D6-dig labeling pattern with Congo red staining using an immunofluorescence method in adjacent brain sections. Congo red dye is known to stain only fibrillar amyloid in parenchymal plaques and in angiopathy (vessel wall). SAR228810-dig mAb mainly labeled focal Aβ deposits that were also stained by Congo red dye, as well as the peri-core area (Fig. 1d). In comparison, 3D6-dig mAb was shown to immunostain not only the Congo red-positive deposits and peri-core area but also a high number of parenchymal diffuse deposits (Fig. 1d). Moreover, no binding was found in human aging healthy brain structures (frontal cortex, calcarine, hippocampus, substantia nigra, olive nucleus, raphe nucleus, cerebellum, and corpus callosum), nor in another 38 types of human tissue that compose the US Food and Drug Administration (FDA) recommended panel, nor in monkey and mouse cross-reactivity studies (data not shown).

In agreement with the biochemical binding selectivity for fibrillar and protofibrillar Aβ, in human AD cortices, SAR228810 specifically immunolabeled Congo red-positive amyloid deposits in angiopathy, peri-vascular deposits, and parenchymal plaques without any labeling in other human organs. In comparison, 3D6 labeled a large number of diffuse Aβ deposits in addition.

#### Protection against oAβ42 toxicity in primary neuronal cultures

**Fig. 2:**
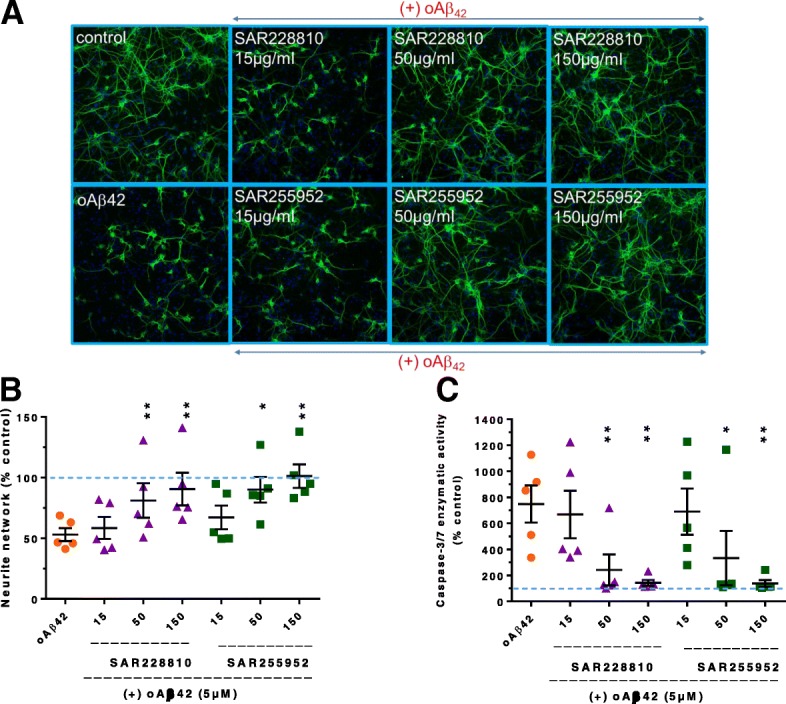
SAR255952 and SAR228810 protect neurons from Aβ oligomer neurotoxicity in vitro. Concentration-response effects of SAR228810 and SAR255952 on the loss of neurite network and the increase in caspase-3/7 enzymatic activity induced by oAβ42 in mouse primary neuronal cultures. **a** Cell images of MAP2 immunostaining in mouse primary neuronal cultures following a 48-h treatment with oligomeric amyloid β (oAβ)42 with or without anti-amyloid antibodies. **b** Quantification of the effects of anti-amyloid antibodies on the neurite network as measured by automated image analysis of MAP2 staining. **c** Effects of anti-amyloid antibodies on cell viability as measured by caspase-3/7 enzymatic activity. *p = 0.0023; **p < 0.0001; *p* = 0.0062; *p* = 0.0352, by two-tailed Dunnett’s test with respect to oAβ42. Horizontal axis represents concentration of respective mAbs in μg/ml. Horizontal black lines and error bars denote mean ± SEM data. Data are expressed as percentage of control group from five independent experiments. Dotted lines correspond to control values without oAβ42 (100%)

The potential neuroprotective activity of SAR228810 and SAR255952 was evaluated in an in-vitro model of oAβ42 neurotoxicity in mouse primary neuronal cultures quantifying neurite network and caspase 3/7 enzymatic activity. Co-treatment of oAβ42 with SAR228810 or SAR255952 inhibited an oAβ42-induced decrease in the neurite network as observed on cell images of MAP2 staining (Fig. 2a). Based on quantitative automated image analysis, SAR228810 and SAR255952 prevented an oAβ42-induced decrease in the neurite network in a concentration-dependent manner, with a maximal protective effect of 80 and 103% at the highest concentration tested (150 μg/ml, i.e., 1 μM), respectively (Fig. 2b). SAR228810 and SAR255952 also inhibited the oAβ42-induced increase in caspase-3/7 enzymatic activity in a concentration-dependent manner with a maximal protective effect of 93 and 94% at the highest concentration tested (150 μg/ml), respectively (Fig. 2c).

In comparison with other clinical anti-Aβ antibodies, SAR228810 had a similar neuroprotective activity when measuring the neurite network compared with gantenerumab and BAN240,1 but was significantly more potent on the caspase-3/7 readout (the latter two being almost inactive) (Table 2). This might suggest that the oβ42 preparation used contained several toxic entities affecting either neurite integrity or caspase activation and that, while SAR228810 can neutralize both, the other two antibodies can neutralize only the former. This adds to the significant differences in binding to the different Aβ conformers observed between the three mAbs. In the absence of oAβ42, no significant effect was observed between control, SAR228810, SAR255952, BAN-2401, or gantenerumab tested alone (data not shown). The isotype control hIgG4 (for SAR228810) or the isotype control mIgG1 and hIgG1 (for SAR255952 and BAN-2401/gantenerumab, respectively) displayed no neuroprotective activity (data not shown). Under these experimental conditions, 3D6-bapineuzumab led to formation of large precipitates with oAβ, preventing evaluation of its neuroprotective activity.

**Table 2:** Comparison of SAR228810 with other clinical anti-amyloid antibodies in neuroprotection assay

% control response (no oAβ42)	oAβ42 5 μM	oAβ42 + SAR228810		oAβ42 + gantenerumab		oAβ42 + BAN-2401	
		50 μg/ml	150 μg/ml	50 μg/ml	150 μg/ml	50 μg/ml	150 μg/ml
Neurite network (MAP2)	49.84 ± 10.29	86.17 ± 10.25	96.49 ± 0.86	70.57 ± 1.66	82.27 ± 6.70	79.34 ± 0.70	86.78 ± 2.75
		*p* < 0.0001	*p* < 0.0001	*p* = 0.0022	*p* < 0.0001	*p* = 0.0059	*p* < 0.0001
Caspase 3/7 activity	291.47 ± 48.73	190.84 ± 39.15	103.42 ± 10.36	388.89 ± 150.02	360.38 ± 123.53	428.48 ± 136.51	355.14 ± 124.57
		*p* = 0.0001	*p* < 0.0001	NS	NS (*p* = 0.2562)	NS (*p* = 0.8675)	NS (*p* = 0.9995)

Control response without oAβ42 = 100%; data are expressed as medians ± median absolute deviation (MAD); *n* = 3–7

*P* values were obtained with a Dunnett‘s test versus oAβ42 after a one-way ANOVA on rank-transformed data

*NS* not significant, *oAβ* oligomeric amyloid β

### Prevention of amyloid plaque development in vivo by murine SAR255952

We next evaluated the efficacy of chronic treatment with murine SAR255952 to prevent brain Aβ deposition in APPSL transgenic mice in comparison with a negative control IgG1, Ctl-IgG1, that recognizes a non-mammalian antigen, and to a reference mouse 3D6, the mouse version of bapineuzumab. Dosing (weekly, i.p.) was initiated at 2 months of age close to the onset of amyloid deposition in this transgenic line.

A 4-month chronic treatment with SAR255952 (10 mg/kg) significantly decreased guanidine-solubilized pan-Aβ levels (−41% at 10 mg/kg, *p* < 0.01) versus Ctl-IgG1-treated controls in the cortex while a nonsignificant trend was observed at 3 mg/kg (−25%) and no effect at 1 mg/kg (+6%) (Table 3). In comparison, the reference 3D6 (10 mg/kg) decreased cortical pan-Aβ levels (−43%, *p* < 0.05) while the dose of 3 mg/kg was inactive (Table 3).

**Table 3:** Antibody dose-dependent prevention of amyloid accumulation and inflammation in APPSL transgenic mice

Treated groups	Ctrl-IgG1	SAR255952			3D6	
Dose (mg/kg)	10	1	3	10	3	10
Biochemical cortical Aβ						
Pan-Aβ μg/g tissue	23.7 ± 1.7	25.1 ± 1.8	17.8 ± 1.7	14.0 ± 1.4	24.3 ± 2.3	13.5 ± 3.4
% reduction vs. Ctrl-IgG1		+6%	–25%	−41%	+3%	−43%
		NS	NS	*p* < 0.05	NS	*p* < 0.001
Aβ42 μg/g tissue	7.62 ± 0.58	8.86 ± 0.74	6.62 ± 0.68	5.28 ± 0.77	8.49 ± 0.84	4.92 ± 1.65
% reduction vs. Ctrl-IgG1		+16%	−13%	−31%	+11%	−35%
		NS	NS	NS	NS	NS
Cystatin F mRNA hippocampus	4.47 ± 0.35	4.27 ± 0.35	2.35 ± 0.29	0.99 ± 0.07	4.99 ± 0.37	2.19 ± 0.49
% reduction vs. Ctrl-IgG1		−4%	−47%	−78%	+12%	−51%
		NS	*p* < 0.05	*p* < 0.001	NS	*p* < 0.05
Aβ immunohistochemistry						
Total area cortex (μm^2^)	382,388	339,950	188,729	83,495	387,344	151,764
SEM	29,945	44,091	19,154	14,776	53,255	31,968
% reduction vs. Ctrl-IgG1		−11%	−51%	−78%	+1%	−60%
		NS	*p* = 0.013	*p* < 0.0001	NS	*p* = 0.003
Total area hippocampus (μm^2^)	103,417	66,849	42,794	20,646	77,838	60,972
SEM	11,020	13,435	4546	4329	9786	8192
% reduction vs. Ctrl-IgG1		−35%	−59%	−80%	−25%	−41%
		NS	*p* = 0.0123	*p* < 0.0001	NS	*p* = 0.0116
Plasma Aβ40 pg/ml	175 ± 31	198 ± 20	201 ± 34	211 ± 27	680 ± 130	999 ± 142

For the different parameters, values represent mean ± standard error of the mean (SEM) unless indicated otherwise

*Aβ* amyloid β, *NS* not significant

SAR255952 treatment did not significantly decrease guanidine-solubilized cortical Aβ42 levels (+16%, not significant at 1 mg/kg; −13%, not significant at 3 mg/kg; −31%, not significant at 10 mg/kg) versus Ctl-IgG1-treated controls (Table 3), nor did the reference 3D6 (+11%, not significant at 3 mg/kg; −35%, not significant at 10 mg/kg). In an independent in-vivo study comparing Ctr-IgG1 and SAR255952 at the dose of 10 mg/kg in a similar 4-month treatment, differential extraction of soluble, membrane, and insoluble fractions demonstrated that SAR255952 led to a comparable decrease in soluble and insoluble fractions of all three Aβ subspecies (Aβ38, Aβ40, and Aβ42) (Additional file 1: Table S2). The membrane fraction interferes in the MSD assay and could not be quantified.

**Fig. 3:**
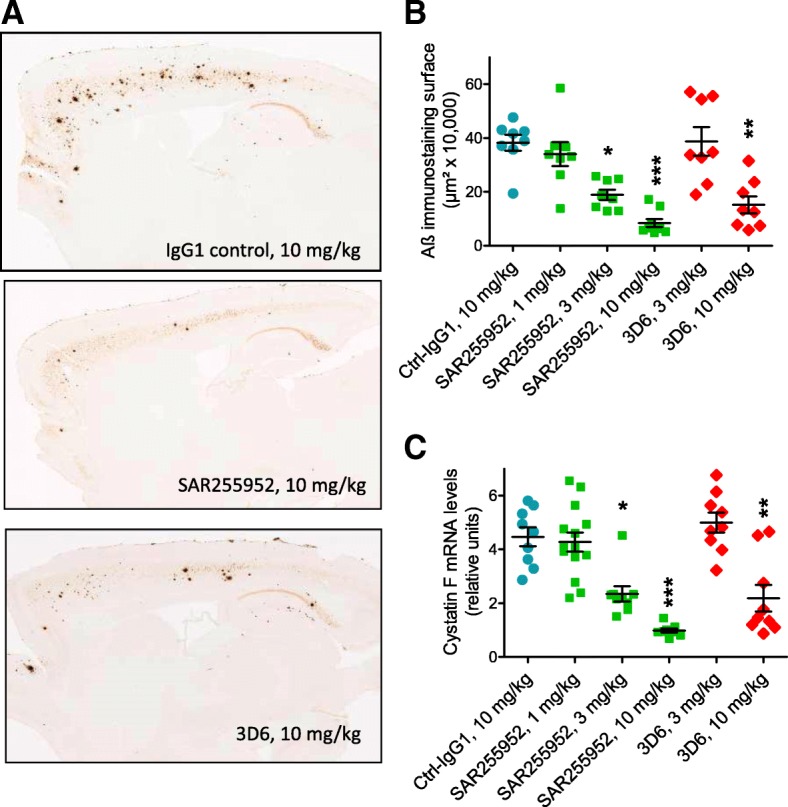
Chronic treatment with SAR255952 prevents amyloid β (Aβ) peptide plaque deposition and brain microglial activation in APPSL transgenic Alzheimer’s mice. Animals were treated for 20 weeks once weekly by the intraperitoneal route with the indicated doses of antibodies starting from the age of 2 months. **a** Whole scanned Aβ-immunostained sagittal brain tissue section illustrating the decrease in the extracellular Aβ peptide deposition process in both cortical and hippocampal brain areas of representative mice chronically treated either with SAR255952 or 3D6, with respect to Ctrl-IgG1-treated mice. **b** Mean values of the total surface occupied by Aβ immunostaining measured over the whole cortical area from eight sections per animal. **c** Real-time PCR amplification of the brain inflammation marker cystatin F expression in the hippocampus. Statistical analysis of both histological and biochemical measurements denotes the significant effect of SAR255952 at 3 and 10 mg/kg injected doses in the cortex. The 3D6 effect is significant at 10 mg/kg. *p < 0.05, **p < 0.01, ***p < 0.001, versus Ctrl-IgG1-treated controls. Horizontal black lines and error bars denote mean ± SEM data

Brain Aβ peptide deposition was further analyzed by immunohistochemistry. All 6-month-old Ctl-IgG1-treated APPSL transgenic mice had developed a reproducible and moderate density of small and large extracellular Aβ deposits over the entire latero-medial axis of both cortical (Table 3, Fig. 3a) and hippocampal brain areas (Table 3).

In SAR255952-treated mice, quantitative analysis evidenced a dose-dependent decrease in the total surface occupied by Aβ immunostaining in extracellular deposits in both cortical and hippocampal brain areas. The decrease in extracellular Aβ peptide deposition in the cortex was −78% (*p* < 0.0001) at 10 mg/kg, −51% (*p* = 0.013) at 3 mg/kg, and 11% (not significant) at 1 mg/kg compared with the Ctl-IgG1-treated control group (Table 3, Fig. 3a). In the hippocampus, the decrease was −80% (*p* < 0.0001) at 10 mg/kg, −59% (*p* = 0.012) at 3 mg/kg, and 35% (not significant) at 1 mg/kg (Table 3). 3D6 treatment at the dose of 10 mg/kg also led to a marked decrease (−60%, *p* = 0.003) in extracellular Aβ peptide deposition in the cortex but was inactive at 3 mg/kg (+1%, not significant, Fig. 3a). In the hippocampus, the decreases were −41% (*p* = 0.012) at 10 mg/kg and −25% (not significant, *p* = 0.089) at 3 mg/kg (Table 2).

From previous characterization of amyloid transgenic models, we selected cystatin F mRNA levels in the hippocampus as a sensitive marker of brain inflammation. SAR255952 treatment significantly decreased cystatin F mRNA levels by −78% at 10 mg/kg (*p* < 0.0001) and −47% at 3 mg/kg (*p* < 0.05) versus Ctl-IgG1-treated controls while, at 1 mg/kg, SAR255952 had no significant effect (Table 3, Fig. 3c). Similarly, 3D6 at the dose of 10 mg/kg significantly decreased cystatin F mRNA levels (−51%, *p* < 0.05) while the dose of 3 mg/kg had no significant effect (+12%, not significant) (Table 3, Fig. 3c). In addition, by immunohistochemistry, the frequency of Iba1-positive activated microglia cells as well as GFAP-positive astrocytes in clusters around Aβ deposits were massively decreased in the SAR255952 high dose-treated animals (data not shown).

Additional characterization by immunohistochemistry revealed that SAR255952 treatment led to a decrease in deposit-associated dystrophic neurites as evaluated with APP immunohistochemistry with antibody 22C11 (data not shown), commensurate with amyloid deposit lowering. Tau phosphorylation levels (AT8 immunoreactivity) at the end of treatment were too limited to be able to document a treatment effect.

These data indicated that a protofibrillar Aβ-specific antibody with much reduced effector functions was at least as effective as a pan-Aβ antibody with full effector function at preventing Aβ peptide deposition and related inflammation.

### Lack of effect of SAR255952 on plasma Aβ levels

Treatment with a range of different anti-Aβ antibodies has been reported to lead to a large increase in plasma Aβ levels, likely due to binding and stabilization of Aβ in the plasma which is otherwise very quickly degraded partly by the liver [41]. At 7 days following the last dose in the chronic treatment study described above, we confirmed that 3D6 treatments leads to a large increase in plasma Aβ40 levels (plasma Aβ42 could not be detected with the sensitivity of our assay), while SAR255952 did not affect Aβ40 levels (Table 3, Additional file 2: Figure S1). At the dose of 10 mg/kg in these samples, SAR225952 levels (149.6 ± 7.9 μg/ml) were higher than for 3D6 (65.5 ± 17.3 μg/ml), indicating that the lack of impact on plasma Aβ40 was not due to lower plasma levels of SAR255952. Such findings are consistent with the specificity of SAR255952 for protofibrillar and fibrillar Aβ forms versus LMW and monomeric conformations present in the circulation.

### Prevention of synaptic activity deficits by SAR255952 treatment

**Fig. 4:**
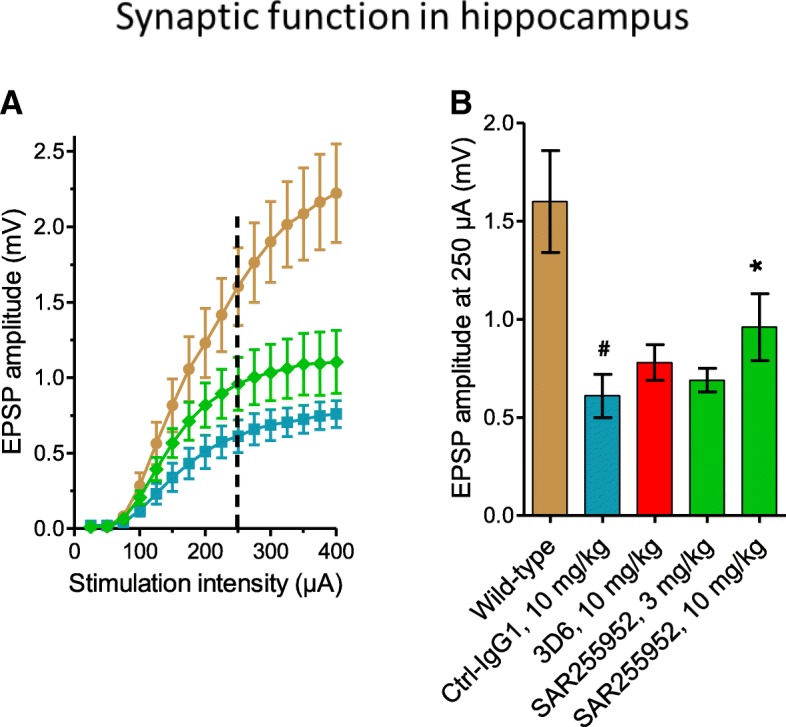
Chronic treatment with SAR255952 prevents synaptic activity deficits in APPSL mice. A subgroup of the transgenic animals was maintained under treatment up to the age of 9–10 months for ex-vivo hippocampal brain slice electrophysiology. **a** Mean (± SEM) size of the extracellular field excitatory postsynaptic potentials (EPSP) recorded in the CA1 stratum radiatum in response to electrical stimuli of the indicated intensity applied to the Schaeffer collaterals of wild-type controls (brown circles), Ctrl-IgG1-treated controls (blue squares), or animals treated with 10 mg/kg i.p. SAR255992 (green diamonds). **b** Mean (± SEM) amplitude of the EPSP at the intermediate stimulation intensity of 250 μA (dotted line on **a**). ^#^*p* < 0.05, versus wild-type controls; *p < 0.05, versus Ctrl-IgG1-treated controls

The functional impact of the prevention of amyloid pathology by SAR255952 was next assessed. Due to the hyperreactivity of the APPSL mouse line and the repeated animal manipulations for treatment (even in the case of weekly administrations), cognitive testing in Object Recognition assay could not be interpreted. Hippocampal synaptic function was evaluated by electrophysiology in brain slices. Treatments with SAR255952 (initiated at 2 months of age) were extended in a subgroup of animals to the age of 9–10 months when strong deficits in synaptic function have developed. In ex-vivo hippocampal brain slices prepared from Ctl-IgG1-treated APPSL transgenic mice, a marked deficit was observed in the glutamatergic synaptic transmission at the level of the CA3-CA1 synapse, the main excitatory pathway in hippocampus. Treatment with SAR255952 prevented the functional alteration to a partial, but significant extent. The protective effect of SAR255952 was visible at all suprathreshold stimulation intensities that elicited measurable field potentials on the stimulation/response curve (Fig. 4a). Measurement of the size of the EPSPs at the intermediate stimulation intensity of 250 μA indicated that APPSL mice chronically treated with Ctl-IgG1 displayed a −62% reduction with respect to wild-type controls (1.6 ± 0.26 mV versus 0.61 ± 0.11 mV, respectively, mean ± SEM, *p* < 0.05, Fig. 3b). Treatment with 3 mg/kg and 10 mg/kg SAR255952 provided a dose-dependent protection against the loss of synaptic function. At the highest tested dose, EPSP sizes reached 0.96 ± 0.17 mV, a significant +35% recovery with respect to Ctl-IgG1-treated animals (*p* < 0.05). In animals treated with the same dose of 3D6 (10 mg/kg), the size of the EPSPs reached 0.78 ± 0.09 mV, a +17% trend for improvement with respect to Ctl-IgG1 controls which did not reach significance (*p* > 0.17, Fig. 4b).

### In-vivo penetration of SAR255952 in brain parenchyma of APPSL mice

**Fig. 5:**
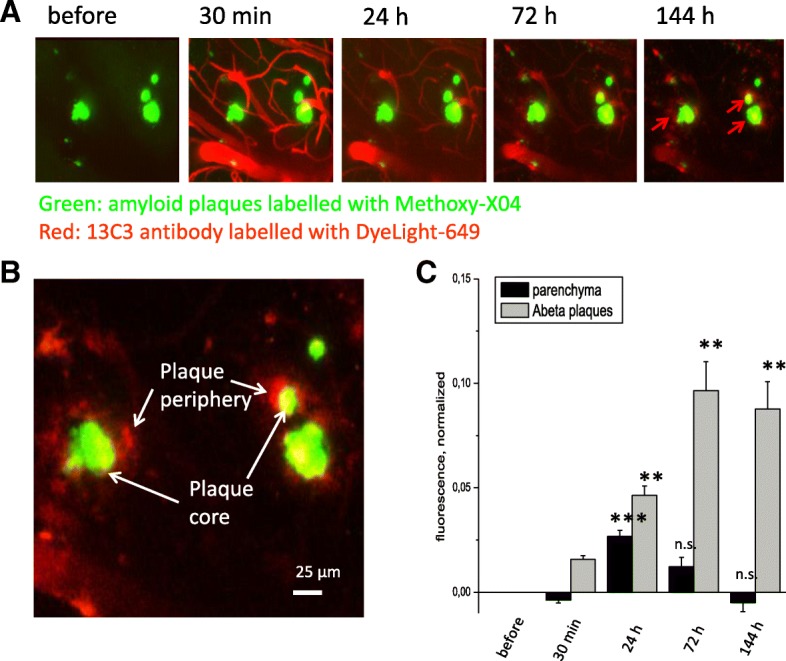
In-vivo penetration of SAR255952 in brain parenchyma and long-lasting binding to amyloid plaques in the cortex of APPSL mice. **a** Successive imaging sessions performed prior to and following the i.v. administration of SAR255952 labeled with Dyelight-649 (red); amyloid plaques labeled by Methoxy-XO4 (green). **b** Higher magnification image showing that SAR255952 (red) decorates Aβ plaques (green) 144 h after i.v. administration. **c** Quantification of temporal changes in the antibody-DyeLight-649 fluorescence showing that SAR255952 accumulates on amyloid plaques while being cleared from the parenchyma. **p <0.01 , ***p <0.005 , versus . n.s. not significant

Using in-vivo two-photon microscopy, the pharmacokinetics of fluorescently labeled SAR255952 was evaluated in three distinct compartments of one female APPSL mouse: 1) brain parenchyma; 2) perivascular deposits; and 3) parenchymal plaques. We found that 30 min after the i.v. injection the labeled antibody was readily detectable in cortical vessels (Fig. 5a). During a follow-up imaging session (24 h after dosing), we observed accumulation of labeled antibody in the brain parenchyma and at the perivascular amyloid deposits. Two additional imaging sessions, performed at 72 h and 144 h after dosing, revealed that the antibody accumulated on parenchymal plaques, while the level of parenchymal fluorescence declined from its peak level observed at 24 h (Fig. 5a, c). Thus, the parenchymal antibody fluorescence was significantly lower than the plaque-associated antibody fluorescence at 72 h and 144 h (*p* < 0.01). This finding is consistent with gradual removal of unbound antibody from the parenchyma and/or redistribution of antibody from the parenchymal compartment onto plaques. Similar in-vivo labeling of amyloid plaques with peripherally administered antibody was also recently demonstrated with another anti-protofibrillar antibody (aducanumab) with no plaque binding with a control IgG [42]. Interestingly, labeled SAR255952 predominantly accumulated on the peripheral parts of parenchymal Aβ plaques (Fig. 5b) and remained there for at least 144 h (7 days), suggesting that chronic weekly treatments might lead to progressive drug accumulation.

**Fig. 6:**
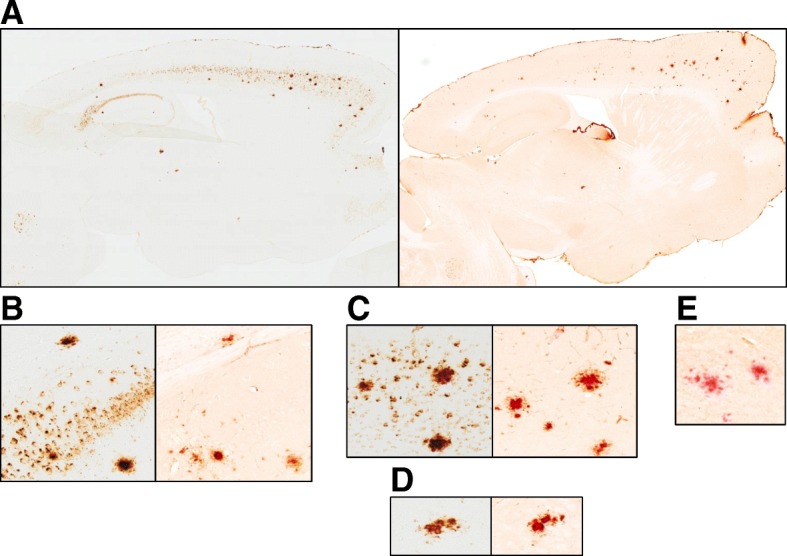
Histological demonstration of SAR255952 brain penetration after chronic peripheral administrations (4-month weekly i.p., 10 mg/kg) in young male APPSL mice. **a** Serial wholly-scanned sagital brain tissue sections with either Aβ (left panel) or IgG1 and Congo red (right panel) immunostaining of one representative animal of the SAR255952-treated group. **b–d** Higher magnification views of individual hippocampal, cortical, and occasional thalamic plaques, respectively showing all remaining plaques with Congo red-positive central core decorated with IgG1 immunostaining. **e** High magnification view of individual plaques in one representative animal chronically treated with Ctrl-IgG1 antibody. Only Congo red- positive central cores of amyloid deposits were detected

Indeed, on brain sections from a 4-month chronic in-vivo study with SAR255952, immunohistochemistry labeling with anti-IgG1 and Congo red histology revealed that most remaining congophilic plaques were decorated with IgG1 labeling (Fig. 6). In contrast, absolutely no IgG1 immunoreactivity was detectable over Congo red stained parenchymal deposits in Ctlr-IgG1-treated animals, as illustrated in Fig. 6e.

Altogether, these data support the concept that systemically administered SAR255952 can cross the blood-brain barrier, diffuse in the parenchyma, and reach amyloid plaques in native tissues, which leads to a progressive accumulation of antibodies on the target.

### Prevention of amyloid plaque development in vivo by humanized SAR228810

Since significant biological differences might exist in the low level of effector functions between a murine aglycosylated IgG1 and a human hIgG4 with an engineered Fc domain, it was important to demonstrate activity of the humanized SAR228810 in vivo and to compare it with the murine version. As adapted from previous reports [39], immunotolerization of APPSL transgenic mice with anti-CD4 transient immunodepletion enabled a 4-month weekly treatment with SAR228810 without immune reaction against the test article; at end of treatment, 1 day following the last administration, plasma levels of humanized SAR228810 (414 μg/ml) were similar to levels of murine SAR255952 (504 μg/ml) while, in non-tolerized mice, SAR228810 plasma levels were minimal after 4-weekly administrations.

**Fig. 7:**
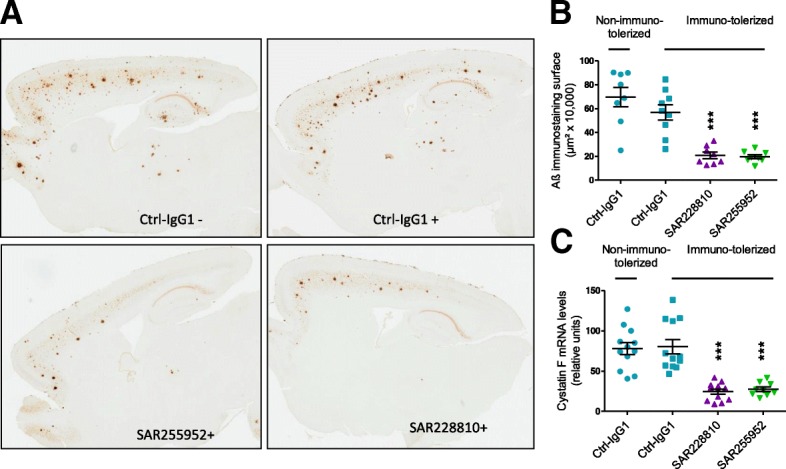
SAR228810, the humanized version of SAR255952, has consistent activity in immunotolerized APPSL mice. Animals were depleted in immunocompetent cells by treatment with a monoclonal anti-CD4+ antibody before undergoing a weekly treatment with 10 mg/kg i.p. of the indicated amyloid β (Aβ) antibodies (labeled + in the micrographs). **a** Representative whole-scanned Aβ-immunostained sagittal brain tissue section illustrating the decrease in the extracellular Aβ peptide deposition process in cortical, hippocampal, and thalamic brain areas in representative immunotolerized mice chronically treated either with SAR255952 or SAR228810, in respect to immunotolerized or not Ctrl-igG1-treated mice. **b** Mean values of the total surface occupied by Aβ immunostaining measured in the cortex over eight sections per animal. The immunodepletion has no significant effect on the cortical Aβ peptide deposition process in Ctrl-IgG1-treated mice. **c** Mean ± SEM real-time PCR amplification readings for the brain inflammation marker cystatin F expression in the hippocampus. Statistical analysis denotes similar significant effects of both SAR228810 and SAR255952. Of note, there was no major effect of the immunotolerization on Aβ peptide deposition in the brain of APPSL mice. **p < 0.01, ***p < 0.001, versus Ctrl-IgG1-treated controls. Horizontal black lines and error bars denote mean ± SEM data

Quantification of brain Aβ peptide deposition by immunohistochemistry confirmed that immunotolerization by itself had not affected Aβ peptide deposition in APPSL transgenic mice. When compared with Ctl-IgG1, treatments with SAR228810 and SAR255952 markedly reduced extracellular Aβ peptide deposition in the cortex (−63%, *p* = 0.0009, and −65%, *p* = 0.0015, respectively, Table 4), in the hippocampus (−67%, *p* = 0.0016, and −73%, *p* = 0.0005, respectively, Fig. 7), and in the thalamus (−83%, *p* = 0.0004, and −71%, *p* = 0.0074, respectively) (data not shown).

**Table 4:** Prevention of amyloid accumulation and microglial activation by SAR255952 and its human homolog SAR228810 in immunotolerized and non-immunotolerized APPSL transgenic mice

	Treatment groups	No. of animals	Mean value	± SEM	Effect size	*p* value
Amyloid β immunohistochemistry total area cortex (μm^2^)						
Non-immunotolerized	Ctrl-IgG1	8	696,471	81,813		
Immunotolerized	Ctrl-IgG1	9	569,092	63,738	−18% vs non-immunotolerized	NS
Immunotolerized	SAR228810	8	207,996	27,252	−63% vs immunotolerized DM4	< 0.001
Immunotolerized	SAR255952	8	197,600	17,064	−65% vs immunotolerized DM4	< 0.001
Cystatin F mRNA hippocampus (related units)						
Non-immunotolerized	Ctrl-IgG1	12	78	7.5		
Immunotolerized	Ctrl-IgG1	12	80	8.9	+ 3% vs non-immunotolerized	NS
Immunotolerized	SAR228810	12	24	3.2	−70% vs immunotolerized DM4	< 0.001
Immunotolerized	SAR255952	9	28	2.8	−66% vs immunotolerized DM4	< 0.001

All animals were treated with the same dose (10 mg/kg) of the indicated antibodies

*NS* not significant, *SEM* standard error of the mean

In the hippocampus, immunotolerization did not affect cystatin F mRNA levels and, compared with control IgG, treatment by SAR255952 or SAR228810 reduced cystatin F mRNA levels by −66% and −70% (*p* < 0.0001), respectively (Fig. 7c, Table 4), confirming the decrease in plaque-associated inflammation.

The humanized IgG4 antibody SAR228810 therefore had a similar activity at preventing amyloid plaque deposition than the mouse aglycosylated SAR255952, strongly suggesting that low to very low effector functions were sufficient to maintain in-vivo efficacy.

### In-vivo safety study with SAR255952 including brain microvessel liability

**Fig. 8:**
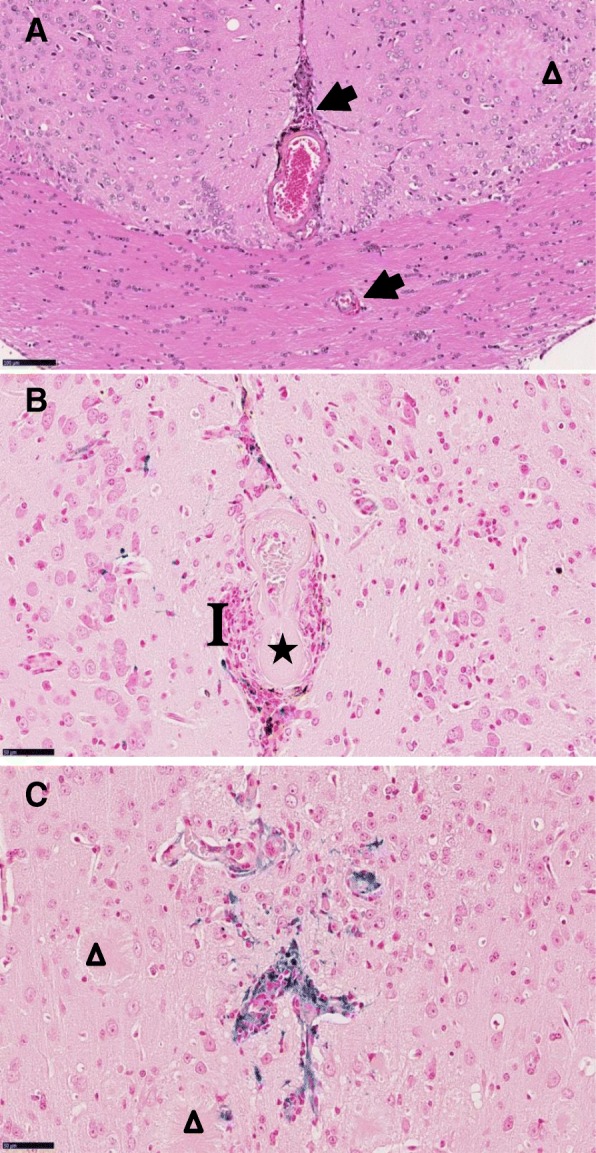
Unlike 3D6, SAR255952 does not cause brain microvessel alterations. Representative histopathological events induced by 3D6 antibody after chronic treatment of 12-month-old female APPSL mice. **a** Perivascular microhemorrhages (arrows) with inflammatory infiltrates and arterial vessel wall degeneration, meningeal and cerebral arteries. Triangles represent amyloid plaques (hemalum, eosin and saffron). **b,c** Periarteriolar iron remnants of hemorrhage in blue. Note arteriolar media hyalinization (*) and perivascular inflammatory cell infiltrates (I) (Perl’s stain for iron with fast red nuclear counterstaining). Scale bars represent 100 μm in panel A and 50 μm in panels **b** and **c**

The main safety concerns with several anti-Aβ mAbs, such as bapineuzumab, gantenerumab, or aducanumab, have been brain vasogenic edema and microhemorrhages (ARIA). It has been suggested that antibody/Fc-receptor binding to microglia and subsequent immune activation at perivascular amyloid deposits are key events in Aβ antibody-mediated microhemorrhages. In the present study, i.v. (bolus) administration of SAR255952 (at doses of 10, 20, or 50 mg/kg) or 3D6 (40 mg/kg) in aged (12–13 months old at initiation of dosing) APPSL transgenic mice once a week for 9 consecutive weeks did not result in any compound-related in-life changes (i.e., mortality, clinical signs, body weight, and food consumption), or any hematology, clinical chemistry, or anatomic pathology findings (i.e., organ weight changes, or macroscopic and microscopic findings). As expected for transgenic APPSL mice of this age, amyloid plaques were noted in all treated animals as rounded, pale eosinophilic, acellular, variably sized areas in the cerebral cortex, hippocampus, and thalamus. Thalamic amyloid plaques were occasionally centered on basophilic material. Minimal brain parenchymal hemorrhagic (Perl’s positive) foci were noted in the brain of mice in the PBS control-and SAR255952-treated groups (with the same incidence and severity). In contrast, in 3D6-treated mice, microscopic vascular changes were detected and consisted of hyalinization of the media in meningeal and/or cerebral arteries associated with degeneration/necrosis of vessel walls in 4 out of 13 mice (Fig. 8a), mild meningeal hemorrhage was observed in the olfactory bulb from the most severely affected animal (data not shown), and an increased incidence of Perl’s-positive staining around vascular structures in association with perivascular inflammation was seen (Fig. 8b, c). Thickening of vessel walls and vasculitis in the vicinity of cerebral amyloid angiopathy-affected blood vessels have also been previously reported [43].

## Discussion

Anti-Aβ immunotherapy represents a major approach in current AD drug development but has had only mitigated clinical success so far. We present here the biochemical and pharmacological properties of a novel antibody, SAR228810, and its murine precursor, SAR255952. They have been selected and engineered to address some of the proposed limitations of current clinical mAbs: 1) specificity for soluble protofibrillar and fibrillar Aβ assemblies/conformations associated with AD pathology to increase efficacy and brain bioavailability; and 2) drastically reduced effector functions to further limit ARIA risks, thereby enabling higher tolerated doses.

Due to its biophysical properties, Aβ peptide (the Aβ40 or Aβ42 isoforms) can form a very broad spectrum of assemblies ranging from monomeric to low and high-n oligomers, soluble protofibrils, and insoluble diffuse deposits to aggregated plaques [11]. However, these different conformations lack a robust biophysical characterization, are highly dependent on experimental conditions, and the presence of some of them in pathological tissues has not been demonstrated [11]. It remains highly debated which Aβ conformation(s) should be neutralized/cleared for therapeutic benefit. Starting with brain pathology studies, soluble oligomeric forms of Aβ have attracted renewed attention. Initially, in case–control studies, brain soluble Aβ levels were correlated with AD dementia [12, 13]. More recently in a series of autopsy cases selected with a similar brain amyloid load, high brain soluble oligomeric Aβ was shown to differentiate demented (CDR > 1) versus non-demented (CDR = 0) cases [14]. Brain soluble oligomeric Aβ was shown to have a strong functional impact, inhibiting acute development of long-term potentiation in vitro and in vivo and leading to neuronal neurite disruption [15–18]. These Aβ high molecular weight soluble species are still under characterization [19, 20] but would represent key candidates to be neutralized for therapeutic benefit.

The binding profile of anti-Aβ mAbs to the different Aβ conformations has been recently reviewed [4] and summarized herein in the Background section. In comparison, SAR228810 binds specifically to soluble high molecular weight protofibrils and fibrillar Aβ versus monomeric Aβ and does not recognize diffuse deposits in human brains.

SAR228810 shows a high selectivity versus monomeric Aβ with no detectable binding to monomeric Aβ by surface plasma resonance and with approximately 100-fold selectivity in ELISA assays, a likely underestimation since monomeric Aβ preparation is subject to early aggregation on adhering to plastic wells in the ELISA format. In comparison, gantenerumab and BAN2401 retain some limited affinity for monomeric Aβ. SAR228810 selectivity versus monomeric Aβ is further confirmed in vivo where it does not increase plasma Aβ levels unlike monomeric Aβ-binding mAs such as bapineuzumab (used here as a positive control), solanezumab, or crenezumab [4] that bind to plasma Aβ and prevents its degradation/clearance in liver [41]. In immunohistology, SAR228810 (up to 5 μg/ml, 35 nM) did not display binding to diffuse Aβ deposits common in elderly cases and which are not related to AD pathology, further documenting that SAR228810 does not bind to Aβ assemblies devoid of β-sheet conformation. In comparison, gantenerumab, in line with its low affinity for monomeric Aβ, has been reported to bind to both diffuse Aβ deposits and compact plaques at 1 μg/ml (7 nM) [8], in addition to cross-reactivity on neurons [44], documenting significant differences in binding profile with SAR228810.

Of interest, SAR228810 neutralizes the neurotoxicity of synthetic oAβ42 in cultures and prevents development of synaptic dysfunction in vivo in amyloid transgenic mice. While the three protofibrillar Aβ mAbs neutralized oAβ42 neurite toxicity, only SAR228810 prevented neuronal apoptosis, further suggesting differences in the binding profiles of the three mAbs across the wide spectrum of Aβ oligomer/protofibril conformers. However, it will be important in the future to compare the binding profiles of those mAbs, including aducanumab, with the different multimeric Aβ assemblies and in particular their capacity to neutralize AD brain-derived synaptotoxic oAβ.

### Brain bioavailability

As previously reported for IgG [45], the murine version of SAR228810 had detectable but low brain penetration, representing approximately 0.03–0.1% of plasma levels. In transgenic mice, 7 days following dosing at 10 mg/kg, SAR255952 concentrations in CSF were in the range of 150–300 ng/ml. In live imaging in mice bearing amyloid plaques, we could document a slow accumulation over days of labeled SAR255952 to the periphery of cerebral amyloid plaques while parenchymal levels decreased and antibody was cleared from plasma. Finally, after chronic treatment in transgenic mice, a strong amyloid plaque-associated IgG1 immunoreactivity could be detected, indicating SAR255952 localization to cerebral plaques.

Due to the measurable but low brain penetration, the antibody selectivity among the different Aβ pools/conformations (which have extremely different ranges of concentrations in the brain as mentioned in the Background section) has a direct impact on its bioavailability in vivo to neutralize/clear each of the cerebral Aβ pathological assembly types. For instance, at the clinical dose of 2 mg/kg, CSF bapineuzumab concentration was 45 ng/ml (0.25 nM) [46], well below the concentration of monomeric Aβ in human CSF (approximately 10 ng/ml, 2.5 nM), and, therefore, most bapineuzumab molecules would be Aβ-bound when reaching the brain parenchyma with limited brain bioavailability to neutralize soluble oligomeric and protofibrillar forms. We would like to emphasize the issue of low therapeutic IgG bioavailability for central nervous system (CNS) indications. It is useful to recall that, for the rare disease amyloid IgG light chain (LC) amyloidosis affecting peripheral organs, the aggregated LC antibody NEOD0001 has recently shown some early signs of efficacy at a monthly dose of 24 mg/kg [47], while IgG penetration in target organs (the kidney and heart) is known to be in the order of 0.1 to 0.5 compared with plasma levels, at least ten-fold higher than for the brain [45]. The recent positive clinical results for aducanumab decreasing brain amyloid in patients were dose-dependent and strongly significant at the highest doses of 6 and 10 mg/kg [7], and similarly for BAN2401 active only at the highest dose (10 mg/kg) tested ([10]), although both had ARIA occurrence. Indeed, the Aβ immunotherapy field is now recognizing this major limitation with a recent trend to largely increase doses when tolerated [48].

The issue of therapeutic antibody bioavailability is further exacerbated by dose limitations due to ARIA adverse effects observed in clinical studies for antibodies binding to vascular amyloid and with effector functions (bapineuzumab from the dose of 2 mg/kg, gantenerumab and aducanumab in clinical studies [26, 49]). To limit the risk of ARIA, SAR228810 was engineered with a double mutant human IgG4 Fc domain to endow drastically reduced effector functions which did not compromise its activity at preventing amyloid plaque development in vivo. Using murine versions, SAR255952 (aglycosylated mIgG1 with very low effector functions) was even moderately more potent than a 3D6 mIgG2a control (murine bapineuzumab) and only minimally less potent than the complete mIgG1 version of SAR255952 (data not shown), the latter two possessing significant high effector functions. Along with amyloid plaque lowering, microglial and astrocyte inflammation were decreased as well as plaque-associated dystrophic neurites, leading to improved synaptic function in the hippocampus. In the animal model used, the impact on cognitive deficit could not be assessed, however. Of note, the human antibody SAR228810 was also shown in vivo to have similar activity as murine SAR255952 using immunotolerized mice. As expected, even at high doses, SAR255952 did not induce brain microhemorrhages and vasculitis while murine bapineuzumab did, similar to its humanized form inducing ARIA in patients.

Unlike in previously reported in-vitro phagocytosis studies, multiple lines of evidence support that effector functions of anti-Aβ mAb are not necessary for activity on amyloid plaques in vivo (discussed in [28]). Using in-vivo multiphoton microscopy, Bacskai and colleagues [50] have demonstrated that 3D6 F(ab’)_2_ fragments (that lack the Fc region of the antibody and therefore effector functions) led to clearance of nearly half of amyloid deposits in APP mice within 3 days, similar to the results obtained with full-length 3D6. These data could be extended to the chronic setting where intraperitoneal chronic treatment with the F(ab’)2 fragment of an Aβ mAb significantly reduced cerebral amyloid plaques, similar to full IgG mAb [51]. Similarly, brain expression of anti-Aβ single chain antibody variable domain (lacking a Fc domain) was also efficacious in amyloid transgenic models [52]. These findings are also consistent with early active immunization data where Aβ immunization was as effective at reducing amyloid deposition in APP mice with FcR-γ chain knockout (FcR-γ^−^/^−^) as in FcγR-sufficient APP mice [53]. Additionally, the deglycosylated anti-Aβ 2H6 (therefore with low effector functions) had an activity comparable to fully glycosylated 2H6 with regard to clearance of brain amyloid plaque and reversing behavioral deficits in the transgenic model [54]. The deglycosylated IgG, however, had the clear advantage of not inducing brain microhemorrhages, similar to that demonstrated in the present report with SAR228810/SAR255952. A similar strategy was adopted for crenezumab which was engineered with a human IgG4 isotype while retaining efficacy in animal studies [55]. A low effector function variant of bapineuzumab (AAB003) developed ARIA-E at higher doses than its parent antibody, confirming that reduced effector function would lower ARIA risk but was discontinued for a lack of effect on biomarkers, suggesting that both epitope and level of effector function could be important [56].

Conceptually, if the therapeutic intent is to neutralize the brain soluble form of synaptotoxic Aβ, there would be a limited requirement for IgG effector function that could even be detrimental in the AD brain which presents a significant inflammatory-primed cerebral environment. Indeed, in aging and in chronic neurodegenerative diseases, modest challenges can lead to more profound CNS inflammation and cognitive deficits than in healthy young individuals, a phenomenon named microglia priming [57]. In acute studies, a full IgG anti-Aβ mAb induced a significant increase in brain immune cells while the corresponding F(ab’)2 fragment did not, while maintaining efficacy on amyloid plaques [51]. Similarly for tau mAbs, it was demonstrated that effector-less IgG maintained efficacy without leading to the neurotoxic activation of microglia triggered by effector-competent IgG [58]. Overall, these results suggest that targeting amyloid in vivo might not require Fc-dependent effector functions.

Regarding the mechanism of action of SAR228810, its binding to soluble protofibrillar Aβ assemblies could prevent further aggregation and synaptotoxicity. In addition, by binding/coating existing plaques, SAR228810 would have the potential to block/prevent secondary Aβ nucleation generated at the plaque surface [59] that could be related to the periplaque protofibrillar Aβ42 “hot spots” associated with axonal dystrophies [60]. It could also prevent leakage from plaques of the oAβ assemblies associated with dementia that have recently been identified in the human AD brain [14]. In this regard, blocking Aβ monomer production, such as with a BACE inhibitor, might be ineffective at preventing the release of toxic species from plaques.

Translating to human AD clinical studies where patients have a high cerebral amyloid deposition many years prior to the onset of symptoms, we are aware that the efficacy of SAR228810 was demonstrated only as a prevention of plaque accumulation and synaptic dysfunction but not for reduction of preexisting amyloid plaque load in the very aggressive APP transgenic line used. There are actually very limited data in the literature demonstrating that in amyloid transgenic mice peripheral administration of an anti-Aβ mAb can decrease preexisting amyloid burden. Even for aducanumab, that has demonstrated a clear dose- and time-dependent decrease of amyloid positron emission tomography (PET) burden in patients, its murine chimeric version could not decrease preestablished amyloid plaque load after systemic administration [42], while being able to prevent plaque accumulation when administered preventatively [7]. The transgenic strains have been designed with very high Aβ production to develop plaques in a few months versus many decades in humans, and only in conditions where Aβ production was stopped or blocked concomitantly could immunotherapy decrease brain amyloid [61, 62]. Of interest in the therapeutic conditions mentioned above, aducanumab improved neuronal calcium levels [42] that could be linked to the cognitive effect in patients, possibly separate from amyloid clearance itself. In the future, it will be important to compare the different high molecular weight Aβ assembly preferring antibodies in more detail and in particular for neutralizing the soluble AD brain synaptotoxic species.

## Conclusions

SAR228810 is a novel antibody with high selectivity for soluble protofibrillar and fibrillar Aβ conformations that has been engineered with drastically reduced effector functions. The expected lack of ARIA in patients could allow us to safely increase antibody doses and, together with the binding selectivity, should lead to a more potent neutralization of brain Aβ species than the antibodies currently in clinical development. SAR228810 and its murine version can neutralize synthetic oAβ neurotoxicity and in animal models prevent amyloid plaque accumulation, related inflammation, and synaptic dysfunction. The potential to block the release/neutralize synaptotoxic Aβ species from amyloid plaques provide rationale for benefit in patients with existing brain amyloid in comparison with small-molecule inhibitors of Aβ production. For clinical studies, SAR228810 target engagement markers such as the capture of high molecular weight soluble Aβ or markers of brain synaptic activity might be more appropriate than amyloid PET. Based on this attractive profile, a first-in-man clinical study has been initiated in AD patients (NCT01485302).

## Additional file

Additional file 1: **Table S1.** Affinity of SAR228810 and SAR255952 for the respective FcγRs. **Table S2.** SAR255952 prevents accumulation of the three Aβ subspecies (Aβ38, Aβ40, and Aβ42) in soluble and insoluble brain fractions in APPSL transgenic mice. (DOCX 42 kb)

Additional file 2: **Figure S1.** Unlike murine bapineuzumab, SAR255952 does not increase peripheral circulating amyloid levels in APPSL transgenic mice. Blood was drawn from animals treated for 4 months once weekly by an intraperitoneal route with the indicated doses of antibodies starting from the age of 2 months. Horizontal black lines and error bars denote mean ± SEM of data. (PPTX 58 kb)

## Abbreviations

ACSF: Artificial cerebrospinal fluid
AD: Alzheimer’s disease
ARIA: Amyloid-related imaging abnormalities
Aβ: Amyloid β
BSA: Bovine serum albumin
CSF: Cerebrospinal fluid
DAB: Diaminobenzimide
DMEM: Dulbecco’s modified Eagle’s medium
DMSO: Dimethylsulfoxide
ELISA: Enzyme-linked immunosorbent assay
EPSP: Excitatory postsynaptic potentials
FDA: Food and Drug Administration
FFPE: Formalin-fixed and paraffin-embedded
FITC: Fluorescein isothiocyanate
GH: Guanidine hydrochloride
HMW: High molecular weight
HRP: Horseradish peroxidase
i.p.: Intraperitoneal
i.v.: Intravenous
IAPP: Islet amyloid polypeptide
IgG: Immunoglobulin G
IHC: Immunohistochemistry
mAb: Monoclonal antibody
MAP2: Microtubule associated protein 2
oAβ: Oligomeric amyloid β
PBS: Phosphate-buffered saline
PBS-T: PBS + Tween
PCR: Polymerase chain reaction
