# Supplementary material for: SAR228810: an antibody for protofibrillar amyloid β peptide designed to reduce the risk of amyloid-related imaging abnormalities (ARIA)
Source: Alzheimers Res Ther. 2018 Nov 28;10:117. doi: 10.1186/s13195-018-0447-y (PMC6264593; doi:10.1186/s13195-018-0447-y)
Supplement: Supplementary file 1 — Table S1. Affinity of SAR228810 and SAR255952 for the respective FcγRs. Table S2. SAR255952 prevents accumulation of the three Aβ subspecies (Aβ38, Aβ40, and Aβ42) in soluble and insoluble brain fractions in APPSL transgenic mice. (DOCX 42 kb) [file 13195_2018_447_MOESM1_ESM.docx]

**Pradier et al, Supplementary Material**

**Table S1 Affinity of SAR228810 and SAR255952 for the respective FcγRs.**

| **Receptor Interaction** | **FcγReceptor (K_D_ nM)** | | | | **C1q (EC_50_ mg/L)** |
| --- | --- | --- | --- | --- | --- |
| **Human Fcγ Receptor** | **hFcγRI** | **hFcγRIIa** | **hFcγRIIb** | **hFcγRIIIa** |  |
| Modulation of immune responses  by hFcγR | activating | activating | inhibitory | activating |  |
| Binding to huIgG (nM)  huIgG1 *[1]*  huIgG4 *[1]*  huIgG4 PE *[2]* | 15  30  very low binding | 200  5000 | 8000  5000 | 500  4000 | +  -  - |
| 3D6-huIgG1  SAR228810 | 0.86  244 | 794  2640 | 2060  1830 | 472  Not measurable | 28  Not measurable |
| **Murine Fcγ Receptor homologue** | **mFcγRI** | **mFcγRIV** | **mFcγRIIb** | **mFcγRIII** |  |
| Modulation of immune responses  by mFcγR | activating | activating | inhibiting | activating |  |
| Binding to mIgG (nM)  mIgG2b*[3]*  mIgG1*[3]* | No binding  No binding | 59  No binding | 448  301 | 1555  3200 | +  - |
| 3D6mIgG2b  SAR255952 | Not measurable  Not measurable | 22  Not measurable | 456  >4500 | 119  Not measurable | 2  Not measurable |
| **Fcγ receptor affinities (K_D_) determined by Surface Plasma Resonance and C1q IC50 by Elisa-type assay. K_D_ reference values from literature provided for two IgG isotypes. Not measurable: up to [mAb] = 6.4 µM; or SPR response was too low to calculate K_D._** | | | | | |

**Table S2** **SAR255952 prevents accumulation the three Aβ subspecies, Aβ38, Aβ40 and Aβ42 in soluble and insoluble brain fractions in APPSL transgenic mice.**

| Fraction/Group | µg/g | Ctrl-IgG1 | SAR2555952 | % inhibition |
| --- | --- | --- | --- | --- |
| Tris Soluble fraction | Aβ38 | 0.160 ± 0.035 | 0.068 ± 0.010 | -58%, p<0.0001 |
|  | Aβ40 | 0.225 ± 0.084 | 0.085 ± 0.025 | -62% p<0.0001 |
|  | Aβ42 | 0.057 ± 0.011 | 0.022 ± 0.003 | -60% p<0.0001 |
| Insoluble fraction | Aβ38 | 25 ± 4.6 | 10.5 ± 2.2 | -59% p<0.0001 |
|  | Aβ40 | 78 ± 18 | 24 ± 5 | -69% p<0.0001 |
|  | Aβ42 | 149 ± 30 | 50 ± 12 | -66% p<0.0001 |
| Initial  homogenate | Aβ38 | 21 ± 8 | 8 ± 3 | -61% p=0.0180 |
|  | Aβ40 | 54 ± 15 | 23 ± 6 | -57% p=0.0120 |
|  | Aβ42 | 46 ± 8 | 21 ± 4 | -55% p=0.0016 |

Values are normalized to the protein content in the initial homogenate. For the different parameters, values represent median ± MAD (Median Absolute Deviation) of Aβ subspecies quantification in cortex samples, n=9 per group

Supplementary references

1. Bruhns P, Iannascoli B, England P, Mancardi DA, Fernandez N, Jorieux S, et al. Specificity and Affinity to Human Fcß Receptors and their Polymorphic Variants for Human IgG Subclasses. Blood. 2009;113(16):3716-25.
2. Reddy MP, Kinney CA, Chaikin MA, Payne A, Fishman-Lobell J, Tsui P, et al. Elimination of Fc Receptor-Dependent Effector Functions of a Modified IgG4 Monoclonal Antibody to Human CD4. J Immunol. 2000;15 164(4):1925-33.
3. Nimmerjahn F, Ravetch JV. Fcγ Receptors: Old Friends and New Family Members. Immunity. 2005; 23:41-51.
