# Supplementary figures and images for: SAR228810: an antibody for protofibrillar amyloid β peptide designed to reduce the risk of amyloid-related imaging abnormalities (ARIA)
Source: Alzheimers Res Ther. 2018 Nov 28;10:117. doi: 10.1186/s13195-018-0447-y (PMC6264593; doi:10.1186/s13195-018-0447-y)

## Slide 1
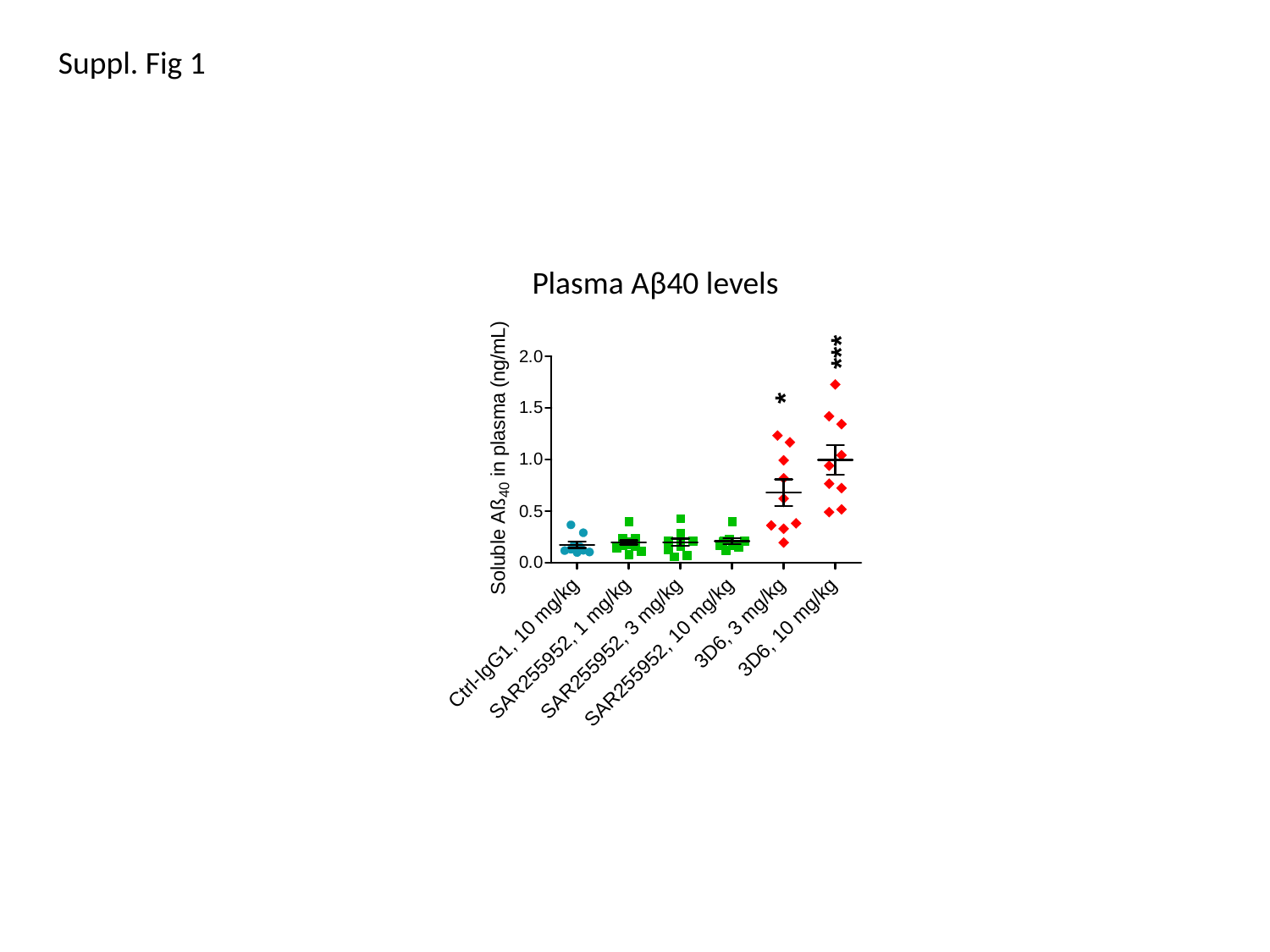

Suppl. Fig 1
Plasma Aβ40 levels

Supplement: Supplementary file 2 — Figure S1. Unlike murine bapineuzumab, SAR255952 does not increase peripheral circulating amyloid levels in APPSL transgenic mice. Blood was drawn from animals treated for 4 months once weekly by an intraperitoneal route with the indicated doses of antibodies starting from the age of 2 months. Horizontal black lines and error bars denote mean ± SEM of data. (PPTX 58 kb) [file 13195_2018_447_MOESM2_ESM.pptx]
